# Coincidence detection in the medial superior olive: mechanistic implications of an analysis of input spiking patterns

**DOI:** 10.3389/fncir.2014.00042

**Published:** 2014-05-01

**Authors:** Tom P. Franken, Peter Bremen, Philip X. Joris

**Affiliations:** Laboratory of Auditory Neurophysiology, Department of Neurosciences, KU LeuvenLeuven, Belgium

**Keywords:** medial superior olive, auditory nerve, input convergence, coincidence window, interaural time difference, interaural correlation, temporal coding, coincidence detection

## Abstract

Coincidence detection by binaural neurons in the medial superior olive underlies sensitivity to interaural time difference (ITD) and interaural correlation (ρ). It is unclear whether this process is akin to a counting of individual coinciding spikes, or rather to a correlation of membrane potential waveforms resulting from converging inputs from each side. We analyzed spike trains of axons of the cat trapezoid body (TB) and auditory nerve (AN) in a binaural coincidence scheme. ITD was studied by delaying “ipsi-” vs. “contralateral” inputs; ρ was studied by using responses to different noises. We varied the number of inputs; the monaural and binaural threshold and the coincidence window duration. We examined physiological plausibility of output “spike trains” by comparing their rate and tuning to ITD and ρ to those of binaural cells. We found that multiple inputs are required to obtain a plausible output spike rate. In contrast to previous suggestions, monaural threshold almost invariably needed to exceed binaural threshold. Elevation of the binaural threshold to values larger than 2 spikes caused a drastic decrease in rate for a short coincidence window. Longer coincidence windows allowed a lower number of inputs and higher binaural thresholds, but decreased the depth of modulation. Compared to AN fibers, TB fibers allowed higher output spike rates for a low number of inputs, but also generated more monaural coincidences. We conclude that, within the parameter space explored, the temporal patterns of monaural fibers require convergence of multiple inputs to achieve physiological binaural spike rates; that monaural coincidences have to be suppressed relative to binaural ones; and that the neuron has to be sensitive to single binaural coincidences of spikes, for a number of excitatory inputs per side of 10 or less. These findings suggest that the fundamental operation in the mammalian binaural circuit is coincidence counting of single binaural input spikes.

## Introduction

The comparison of sound waveforms at the two ears is a prime model for temporal processing in the central nervous system. The underlying circuit is of wide interest because it implements a neural analog to crosscorrelation, and is well-suited to study coincidence detection. Humans are exquisitely sensitive to two dimensions of binaural temporal differences: interaural time differences (ITDs) and interaural correlation (ρ) (Durlach and Colburn, 1978; Trahiotis et al., 2005). ITD is the dominant cue for horizontal sound localization. Sensitivity to rho is important to hear out signals in noise and for spatial hearing in complex acoustic environments such as concert halls.

In mammals, the processing of binaural temporal differences starts in the medial superior olive (MSO) (Joris and Yin, 2007; Grothe et al., 2010), which is the only site where monaural neurons of the same cell class converge from the left and right side onto single neurons. The excitatory input to these neurons is provided by spherical bushy cells (SBCs) in the cochlear nucleus (Cant, 1991); inhibitory input is derived from globular bushy cells (GBCs) through relays in the medial and lateral nuclei of the trapezoid body (Cant and Hyson, 1992). SBCs and GBCs encode the fine-structure of the sound up to a few kHz with high precision and reliability (Joris et al., 1994a,b; Louage et al., 2005). By comparing the timing from both ears, MSO cells are sensitive to differences in ITD and ρ (Goldberg and Brown, 1969; Yin and Chan, 1990). This is thought to be based on coincidence detection (Jeffress, 1948; Goldberg and Brown, 1969; Yin and Chan, 1990). Unfortunately, there is little data from MSO, due to large field potentials (Mc Laughlin et al., 2010; Day and Semple, 2011) and intrinsically small action potentials (Scott et al., 2007). This is particularly the case for responses to non-tonal stimuli. Several issues remain unresolved regarding the precise nature of this coincidence process. A first issue concerns the degree of convergence. Coincidence detection is usually portrayed as a process in which coincidences of individual input spikes trigger postsynaptic spikes. However, in nucleus laminaris of the barn owl (NL, a binaural structure homologous to MSO), a vast number of inputs generate summed post-synaptic potentials that result in an intracellular analog voltage pattern resembling the acoustic waveform (Funabiki et al., 2011; Ashida et al., 2012): binaural processing in this structure is more akin to a process of phase coherence detection than one of coincidence detection. The number of inputs converging on MSO neurons is not known but is thought to be low (Couchman et al., 2010), and there is significant variability in the cycle-to-cycle subthreshold responses (Franken et al., 2013; van der Heijden et al., 2013). However, it is still unclear whether output spikes are generated in response to single input spikes, or whether temporal summation of multiple inputs on each side is required. A second issue is that multiple inputs from one side result in monaural coincidences, which can interfere with binaural sensitivity. Colburn et al. (1990) suggest that monaural coincidences inherently have much lower probability due to the lower number of permutations generating coincidences across monaural than across all input fibers (see Results). On the other hand, it has been suggested that the bipolar shape and dendritic segregration of monaural inputs generates a nonlinearity favoring binaural coincidences (Agmon-Snir et al., 1998).

A third issue is that the simplest model of coincidence detection, applied to spike trains recorded from SBCs, results in outputs that deviate significantly from actual binaural responses (Mc Laughlin et al., 2008). In that work, a simple coincidence analysis was applied to responses of single axons in the trapezoid body (TB), originating from SBCs and GBCs, as well as to responses of auditory nerve (AN) fibers. Since the main tool in that analysis was the autocorrelogram, we refer to it as the AC analysis. The dependence of number of coincidences on binaural parameters showed fundamental similarities to the spike rate in actual binaural responses. However, the spike rates were unphysiologically low. Furthermore, this dependence was surprisingly more acute (i.e. with a steeper dependence on ITD or interaural correlation) for coincidences calculated from TB fiber responses than for real binaural responses. A possible explanation for the shallower dependence on ITD and interaural correlation of binaural neurons than expected, suggested to us by Dr. Torsten Marquardt (personal communication), is an inherent “compressive effect” in a coincidence process with many inputs (as opposed to the single inputs used in previous AC analyses). Indeed, in a simple coincidence scheme it does not matter which spikes coincide among all the possibilities, and only one output spike can be generated irrespective of the number of input spikes coinciding. This may decrease changes in firing rate for interaural correlation values close to 1, or ITD close to the best delay, and therefore make the resulting functions less steep. We refer to this suggestion of an inherent compression between monaural input and binaural output as “binaural compression.”

Modeling studies can give insight in the process of coincidence detection in MSO, particularly in view of the difficulties in recording from these neurons. Considering the limited experimental data, a surprisingly large number of MSO models has been published (Colburn et al., 1990; Agmon-Snir et al., 1998; Brand et al., 2002; Zhou et al., 2005; Jennings and Colburn, 2010; Leibold, 2010; Fischl et al., 2012; Sanda and Marsalek, 2012; Brughera et al., 2013). These models reproduced ITD functions reasonably well. However, one limitation of these models is that the inputs were only rarely modeled on plausible SBC spike trains (Brughera et al., 1996). Another limitation is that none of them made predictions of responses to broadband noise. For ITD-sensitive neurons in the inferior colliculus (IC), responses to broadband noise are much more tightly linked to the physiological properties of the neurons than responses to tones (Yin et al., 1986; Joris, 2003; Joris et al., 2005), and this is likely also the case in the MSO (Yin and Chan, 1990). For example the sign of binaural interaction; frequency tuning; and the relative sensitivity to fine-structure vs. envelope can more readily and reliably be extracted from responses to broadband noise. Responses to noise are therefore more constraining for a model than responses to tones.

Unlike previous models of increasing sophistication, we use a bare bones approach to examine to what extent coincidence patterns of realistic input spike trains in response to broadband noise are able to result in actual binaural responses. As inputs we use noise responses recorded from TB fibers and AN fibers of cats. We extend the AC analysis by varying the number of inputs per side, the efficiency of monaural and binaural coincidences and the coincidence window. Coincidence detection is implemented using a simple scheme of counting coincidences across inputs. Individual noise responses from the same fiber are used as separate inputs to the coincidence detector. The output is based on the simple assumption that an output spike can only be generated if two or more input spikes occur close enough in time. By comparing the output to data recorded from binaural cells in the IC or the MSO, we evaluate which parameter values result in physiologically plausible output. Our simple approach does not address cellular details of the process of coincidence detection, but provides several new insights into this process, given the properties of the input spiketrains recorded *in vivo*.

## Materials and methods

SBCs and GBCs receive input from auditory nerve (AN) fibers and project their axons into the trapezoid body (TB). For comparison with previous modeling studies (see Introduction) and to evaluate the effect of the transformation of spike output in the cochlear nucleus, we perform the simulations with TB as well as AN fiber data.

We present analyses of archival TB and AN recordings. The procedure of generating pseudobinaural noise delay functions and interaural correlation functions with multiple unilateral inputs was briefly described for one TB fiber in (Mc Laughlin et al., 2014).

### Recording

Fiber responses were obtained in cats (*N* animals = 5 for TB data and 2 for AN data) under general anesthesia. All procedures were approved by the K.U. Leuven Ethics Committee for Animal Experiments and were in accordance with the National Institutes of Health Guide for the Care and Use of Laboratory Animals. The recording procedures have been described before (Louage et al., 2005, 2006). In short, cats with normal eardrums and middle ears were brought under anesthesia with acepromazine (0.2 mg/kg) and ketamine (20 mg/kg). Sodium pentobarbital i.v. was used for maintenance of anesthesia. The animal was placed on a heating pad in a double-walled soundproof room. The pinnas were removed and the bullas exposed and vented. For TB experiments, the basioccipital bone was exposed and a longitudinal slit drilled close to the medial wall of the bulla. Since the TB contains crossing fibers, both ipsi- and contralateral fibers can be recorded from a single location. The AN was exposed via a posterior fossa craniotomy. Glass micropipettes filled with 3M NaCl or KCl were positioned in the TB or AN under visual control. Sounds were presented through speakers attached to ear bars that were tightly inserted into the ear canals. Sound stimuli were compensated for the acoustic transfer function measured with a probe tube near the ear drum. The neural signal was amplified, filtered and the action potentials were timed with 1 μs resolution.

### Stimuli

The characteristic frequency (CF) of single fibers was determined using an automated threshold-tracking routine. Responses to short tone bursts at CF were obtained to allow classification of the fiber according to the shape of the peristimulus time histogram (Pfeiffer, 1966; Smith et al., 1991, 1993). Broadband noise stimuli were used with different inter-token correlation values. These were generated by mixing independent tokens of broadband noise with the same bandwidth (Robinson and Jeffress, 1963; Louage et al., 2006). The noise had a high-pass cut-off frequency of 50 or 100 Hz and a low-pass cut-off frequency between 8000 and 32000 Hz. Inter-token correlation values were (1; 0.99; 0.96; 0.91; 0.84; 0.76; 0; −1). Sound intensity was 70 dB SPL. The number of repetitions obtained per noise token varied from 20 to 75. Stimulus duration was either 600 ms (most AN fibers) or 1000 ms (all TB and some AN fibers) and interstimulus interval was between 1000 and 1400 ms.

### Selection of fibers for analysis

Only fibers with a CF up to about 1500 Hz were selected, which covers the range important for fine-structure-based sensitivity to ITDs and enhanced synchronization of TB fibers relative to AN fibers (Joris et al., 1994a; Joris, 2003; Louage et al., 2005). For the TB fibers, we further limited the sample to fibers showing a “phase-locked” or “primary-like” type of response in the peristimulus time histogram. Fibers with a “primary-like” type of response are most likely to be SBCs, and fibers with a “phase-locked” type of response can either be SBCs or GBCs (Smith et al., 1991, 1993). The goal of this selection was to increase the proportion of SBCs as much as possible in our sample of TB fibers, since these are the excitatory inputs onto MSO neurons. With these constraints, 15 TB fiber datasets (CF: range 230–1518 Hz) and 16 AN fiber datasets (CF: range 136–1306 Hz) were selected for the current analysis.

### Binaural coincidence model

In order to simulate a binaural coincidence response to broadband noise, we used responses of a monaural (AN or TB) fiber to broadband noise as “ipsi-” and “contralateral” inputs to the binaural cell. These responses mimicked the input of several SBCs onto one MSO cell for a particular binaural noise stimulus.

The first step in the simulation was to select input spike trains. For each neuron recorded, each stimulus was repeated *N_tot_* times. In order to simulate a single “run,” we randomly selected a number of spike trains (*N*) as ipsilateral inputs, and an identical number of different spike trains as contralateral inputs. The ipsi- and contralateral spike trains could be in response to the same stimulus (correlated noise), e.g., when calculating a noise delay function (NDF), or to different stimuli (partially correlated or uncorrelated noise), e.g., when calculating a rate interaural correlation function (rICF). A given spike train was never present more than once in a single run of a binaural simulation. For a given simulation, all spike trains were responses of the same fiber. Note that once they are pooled, the monaural input spike trains for each side lose their “identity”: the input now consists of a collection of spike times which are treated equally without regard to their origin.

**Figure 1:**
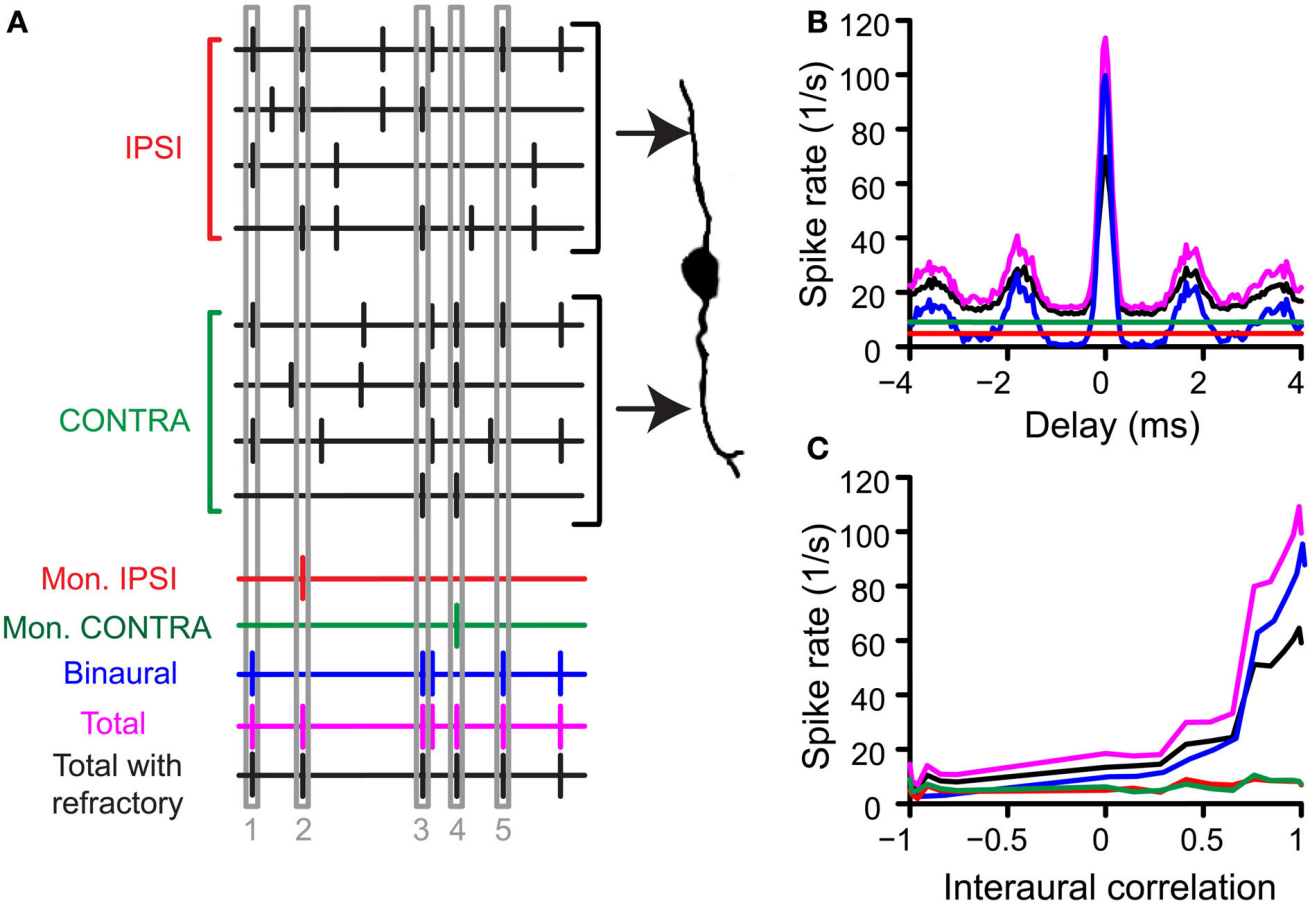
**Simulation of coincidence detection. (A)** Depiction of one run of the coincidence process. In this example, *N* = 4, *thr_mon_* = 3, *thr_bin_* = 2 and *cw* = 50 μs. See text for details. **(B)** Example of pseudobinaural NDF. The traces shown are the average rates of 3 real runs with the same parameters as in **(A)**. Colors correspond to those of the spike trains in **(A)**. Data from a TB fiber with a CF of 559 Hz (SR 1.27 spikes/s). **(C)** Example of rICF, obtained by choosing responses to noise tokens with different intertoken correlations as inputs on the “ipsilateral” and “contralateral” side. Same fiber and parameters as in **(B)**.

In a second step of the simulation, the operation of the binaural cell was simulated by counting coincident input events, i.e., events occurring within the same predefined time interval, the coincidence window (*cw*), centered at spike occurrence. This process of counting coincidences is illustrated in Figure 1A. In this example the number of inputs is 4 per side. From a collection of spike trains recorded from one (TB or AN) fiber in response to the same noise, 4 spike trains have been randomly selected as “ipsilateral” inputs, and 4 other spike trains as “contralateral” inputs. Then, coinciding spikes are counted across the input spike trains of one side (monaural coincidences) and across input spike trains from both sides (binaural coincidences). This is done by grouping all spike times from all relevant spike trains (monaural or binaural), sorting them in time, and counting, for each spike, the number of following spikes that fall in the same *cw*. We used a monaural coincidence threshold (*thr_mon_*) and a binaural coincidence threshold (*thr_bin_*) to decide whether respectively monaural or binaural coincident input spikes are effective (result in output spikes). If the number of coinciding spikes for one unilateral group of spike times exceeded *thr_mon_* (set at 3 in the example in Figure 1A), a monaural coincidence was counted, and timed at the last coinciding spike in that window. In Figure 1A, there is one effective monaural coincidence for the ipsilateral inputs (red, case 2, indicated by numbers at the bottom) and one effective monaural coincidence for the contralateral inputs (case 4, green). Similarly, if the total number of coinciding spikes in the total (binaural) pool of spike times exceeded *thr_bin_* (set at 2 in Figure 1) and each side contributed at least one spike, a binaural coincidence was counted (blue trace). This coincidence was again timed at the last coinciding spike. The total output spike train was calculated by adding (logical “OR” operator) the monaural and binaural coincidences (Figure 1A, magenta trace).

Note that these steps contain the inherent compression mentioned in the Introduction: only one effective coincidence could be counted per *cw* (monaurally and binaurally) even if there are multiple coincidences. For example, in the fictional example shown here, the binaural output is 1 for a bin with 2 coincident spikes (case 5) as well as for one with 4 coincident spikes (case 1). Note that *cw* was always equal for monaural and binaural coincidences (see also Discussion).

The total output spike train for many runs (not shown) gave a probability density function of coincidences for a given AN or TB fiber but could contain unphysiologically small intervals. We therefore imposed a refractory period of 1 ms by sequentially running through the total spike train and removing all spikes that occur within 1 ms after the previous spike. The length of the refractory period was determined by evaluating the interspike interval (ISI) histogram of noise responses from chinchilla lateral lemniscus (LL) fibers [Figure 3F, data from (Bremen and Joris, 2013)]. Only LL fibers where the number of false triggers—defined as ISIs < 0.5 ms—was below 0.01% were included in Figure 3F. The result after removal of “refracted” spikes was the final output (black trace). As is standard in physiological analyses, we summed the total number of coincidences or “spikes” over the stimulus duration and expressed this number as a rate (“spike rate” in spikes/s). This spike rate was used in all further analyses unless specified otherwise. The entire procedure was then repeated with newly drawn spike trains. We repeated this process three times for each choice of parameters and averaged the output spike rates.

We varied the following parameters of the coincidence process: the number of inputs per side (*N*), the monaural coincidence threshold (*thr_mon_*), the binaural coincidence threshold (*thr_bin_*), and the duration of the coincidence window (*cw*). Unless stated otherwise, *N* was varied from 1 to 10 (which covers the presumed range of MSO inputs per side, see Discussion), *thr_mon_* from 2 to 11, *thr_bin_* from 2 to 11 and *cw* was 50, 150, or 250 μs.

### Noise delay functions

From the responses to noise, we computed two “pseudobinaural” functions which together provide a characterization of the influence of two basic binaural parameters: ITD and interaural correlation (Mc Laughlin et al., 2008). By time-shifting the “ipsilateral” relative to the “contralateral” inputs different ITDs were mimicked. When the output was represented as spike rate, a pseudobinaural noise delay function (NDF) was generated (Figure 1B). In addition to a NDF to correlated noise, where ipsi- and contralateral input spike trains were taken from responses to the same noise token, we also obtained a NDF to anticorrelated noise i.e., where the responses of one side are in response to the same noise waveform as on the other side but inverted in polarity. Obtaining responses to two opposite polarities is a standard procedure in our laboratory: it allows a fuller description and analysis of both monaural and binaural responses (Joris, 2003; Louage et al., 2004).

### Rate interaural correlation functions

A second fundamental binaural parameter is ρ (Blauert, 1970; Yin et al., 1987; Albeck and Konishi, 1995; Shackleton et al., 2005; Coffey et al., 2006; Louage et al., 2006). To simulate interaural correlation sensitivity, we used spike trains in response to noises that had varying degrees of correlation. Responses were paired in all permutations possible (of the 8 noise tokens presented) (Louage et al., 2006). This was always done at an ITD of 0 ms. Plotting the output spike rate as a function of ρ resulted in a pseudobinaural rate-interaural correlation function (rICF) (Figure 1C).

### Validation of model output by comparison to binaural data

For each fiber and for each set of model parameters we calculated a NDF (for correlated and anticorrelated noise) and a rICF. To explore the parameter space, we assessed the physiological plausibility of the final output functions (corresponding to the black trace in Figures 1B,C) by comparing their features to data from actual binaural cells. The features that were examined for physiological plausibility are spectral bandwidth (BW) and dominant frequency (DF); the steepness of the rICF; the maximal rate of the NDF (peak rate); the ITD modulation of the NDF and the halfwidth of the main peak of the NDF. For each of these features we bracketed the range of acceptable values, based on the same features in data from actual binaural responses as obtained in the cat IC (Mc Laughlin et al., 2008) or in axons from presumed MSO neurons of chinchilla (Bremen and Joris, 2013). A particular simulation was only accepted if it passed all “acceptance criteria.” Below we describe every acceptance criterion. Collectively they allowed us to identify model parameters that generate plausible output. We tested a range of criteria: none of our conclusions critically depend on the details of the definition of criterion boundaries.

#### BW and DF

**Figure 2:**
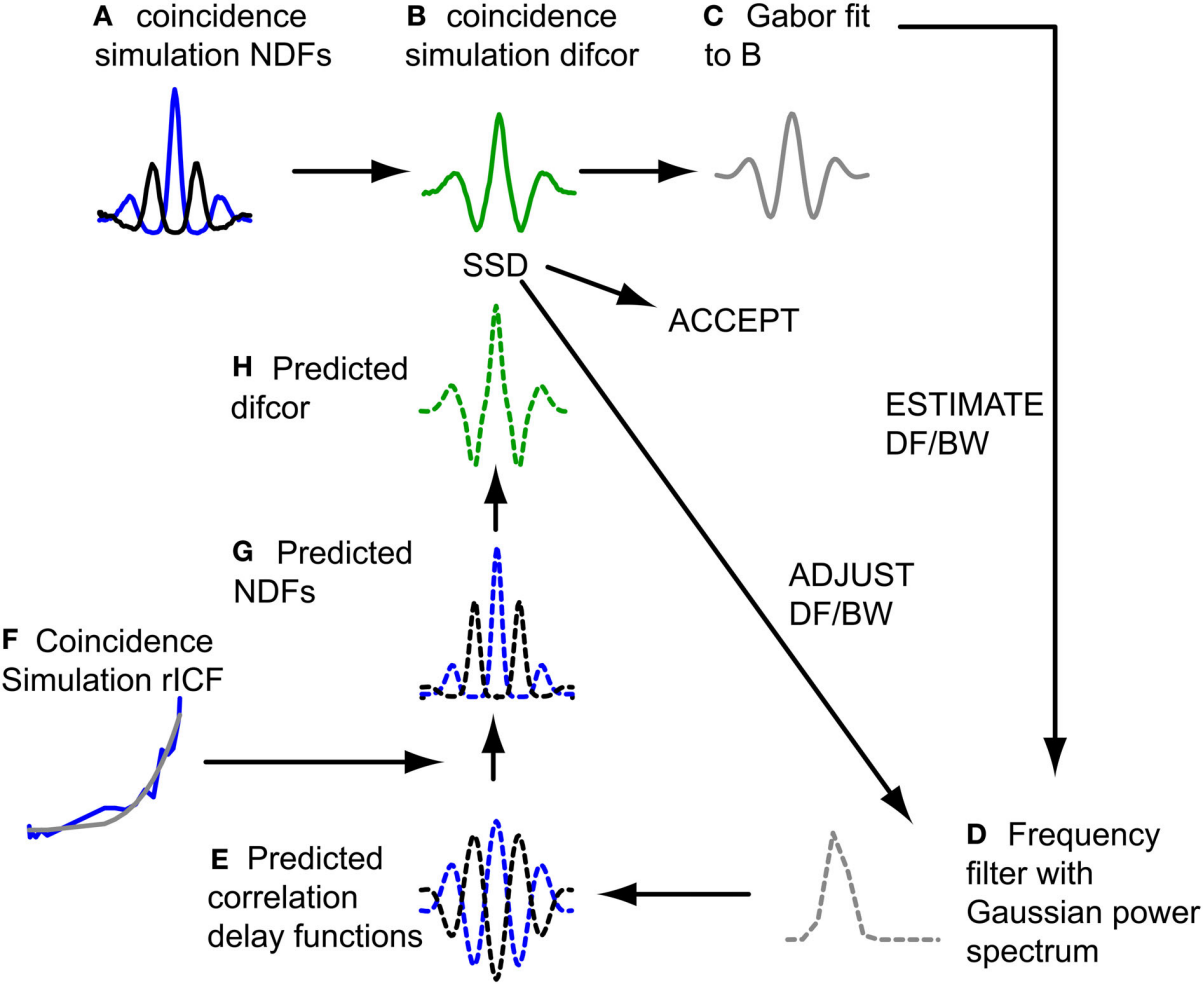
**Fitting procedure of NDFs to estimate DF and BW.** Dashed lines indicate filter model. Blue lines represent ρ = 1, black lines represent ρ = −1. See text for detailed explanation of steps **(A–H)**.

The filtering action of the cochlea restricts the spectral bandwidth of a broadband sound that affects a binaural neuron, and the response of these neurons is usually modeled as a time domain crosscorrelation operating on the filtered sound. For the CF range considered here (see Selection of Fibers for Analysis), the effect of spectral filtering is to give the NDFs a damped oscillatory shape. The exact shape is not only determined by peripheral filtering, but also by the (typically non-linear) relationship between interaural correlation and spike rate, which can be quantified with the rICF. Thus, in combination, the NDF and rICF allow us to estimate the center frequency and bandwidth of the cochlear filtering that determines a neuron's binaural temporal sensitivity. Previous work from our lab has measured these two properties for IC neurons via a fitting procedure (Mc Laughlin et al., 2008), based on a method used for psychoacoustically measured NDFs (van der Heijden and Trahiotis, 1999), and found a clear relationship between center frequency and bandwidth (BW). Here we used a similar approach to determine corresponding values for the coincidence simulation results, and then check whether these values are within the range obtained for actual binaural neurons (Mc Laughlin et al., 2008). Figure 2 illustrates the procedure.

The difcor for a particular simulation output (Figure 2B) was obtained by subtracting the NDF for anticorrelated stimuli from the NDF for correlated stimuli (Figure 2A). Next a Gabor function was fitted to the difcor (Figure 2C). The oscillation frequency and bandwidth of this fit defined a frequency filter with a Gaussian power spectrum (Figure 2D): the mean of the spectrum (center frequency) corresponded to the oscillation frequency of the Gabor fit, and its standard deviation to half of the bandwidth of the Gabor fit. Group and phase delay of the filter were 0. Indeed there was no interaural delay (and more generally no filter differences at all) in our coincidence model because responses from the same neuron, at the same SPL, provided both contra- and ipsilateral inputs. To simulate the case of anticorrelated noise, we used a copy of the filter for which the phase delay was π. Inverse Fourier transformation of the filter transfer functions resulted in predicted correlation vs. delay functions (Figure 2E). These could not be compared directly to the pseudobinaural NDFs, since spike (or coincidence) rate is not always linearly related to correlation either in actual binaural responses (Yin et al., 1987; Albeck and Konishi, 1995; Saberi et al., 1998; Shackleton et al., 2005; Coffey et al., 2006) nor in pseudobinaural functions (Louage et al., 2006; Mc Laughlin et al., 2008). The correlation vs. delay functions was therefore first transformed to predicted spike rate vs. delay functions (NDFs) (Figure 2G) using the rICF (Figure 2F). The predicted NDF for anticorrelated noise was then subtracted from the predicted NDF for correlated noise to obtain the predicted difcor (Figure 2H). The sum of the squared differences (SSD) between this predicted difcor and the difcor of the coincidence simulation (Figure 2B) was minimized using a built-in function in Matlab (lsqcurvefit), by changing DF and BW and looping through steps D–H until an adequate fit was reached. The quality of the fit was described by the quality factor (*Q*), that described the fraction of variance accounted for by the fit,

(1) $$Q=1-\frac{\sum_{k}{\left(Y_{fit}{}_{k}-Y_{data}{}_{k}\right)}^{2}}{\sigma_{data}^{2}}$$

where *Y_fit_* was the predicted difcor for *k* interaural delays, *Y_data_* was the difcor from the coincidence simulation and σ^2^_data_ was the variance of the difcor from the coincidence simulation.

**Figure 3:**
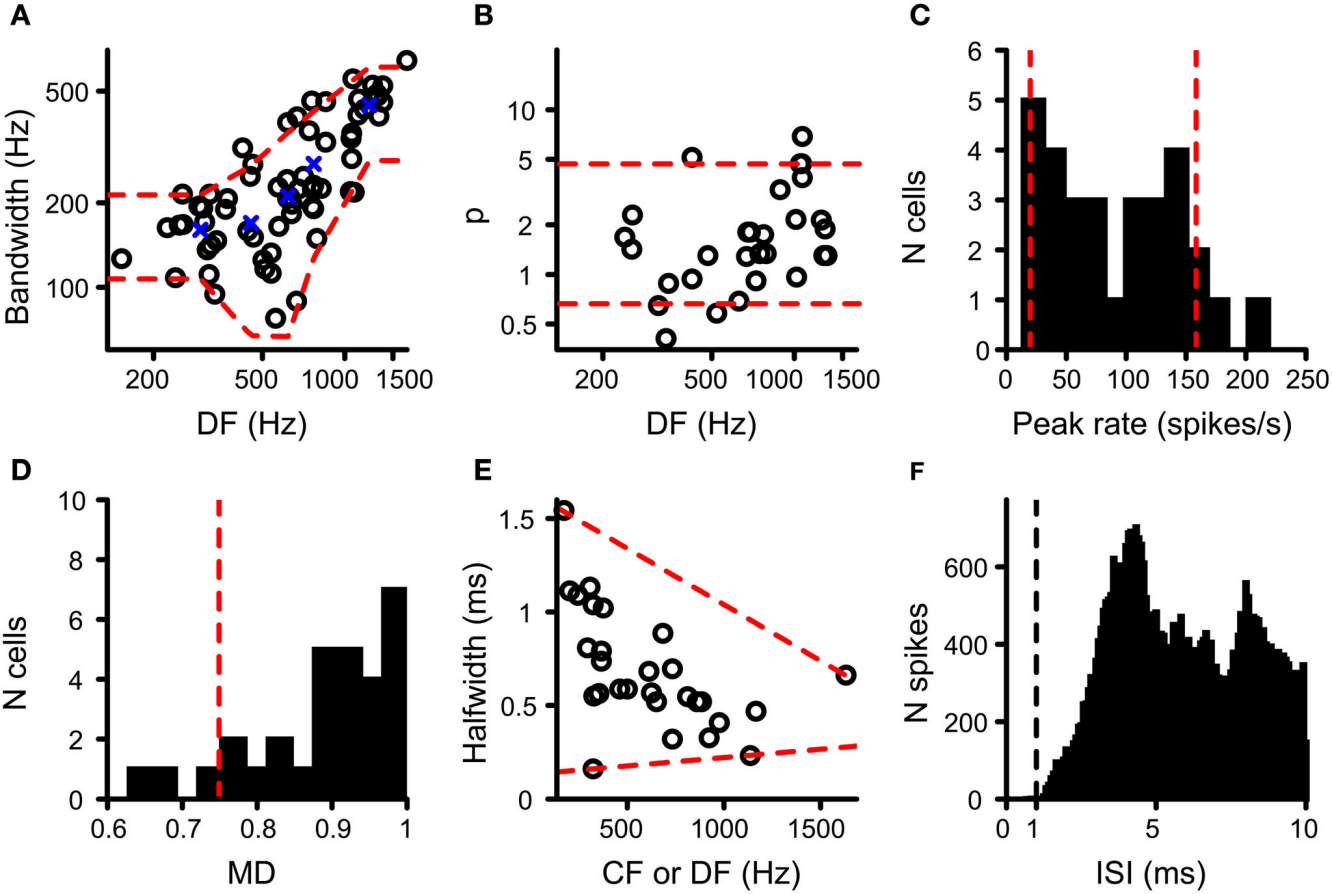
**Data from binaural cells used to constrain simulation output.** Red dashed lines indicate the parameter range accepted for coincidence simulation output (see text for details). **(A)** Scatter plot of bandwidth vs. DF for cat IC datasets (circles; *n* = 68). **(B)** Power of rICF as a function of DF for cat IC (*n* = 29). Data in **(A)** and **(B)** taken from (Mc Laughlin et al., 2008, 2014). **(C)** Histogram of peak firing rate of chinchilla LL noise delay functions (*n* = 30). **(D)** Histogram of modulation depth of the same chinchilla LL datasets (*n* = 30). **(E)** Scatter plot of halfwidth of central peak of noise delay function as a function of CF or DF, for chinchilla LL fibers (*n* = 28). **(F)** ISI histogram for chinchilla LL fibers (*n* = 35). Only units where the proportion of spikes with ISI < 0.5 ms is smaller than 0.01% of the total number of spikes are included. Vertical dashed line indicates the chosen refractory period in the coincidence counting scheme. Data in **(C–F)** from Bremen and Joris (2013).

The resulting values for DF and BW were accepted when *Q* was at least 0.7 and the pair of (DF, BW) values was within the range observed in binaural cells in the cat IC (*N* = 68) (Mc Laughlin et al., 2008). The red dashed lines in Figure 3A indicate the upper and lower limits of acceptance that are relevant to the cells tested here. These limits were obtained by calculating, for the cat IC datasets (circles in Figure 3A), the mean BW for five DF bins where the center of these bins are the quantiles 10%; 30%; 50%; 70%; 90% of the IC DF values (blue crosses in Figure 3A). Then for each bin the mean BW ± 1.5 *SD* was calculated, which when connected resulted in the red dashed lines in Figure 3A. The DF and BW obtained for a coincidence simulation had to be within these limits for the simulated coincidence output to be accepted.

#### rICF

An important motivation for the current work was to tackle the question whether convergence of multiple inputs would decrease the steepness of the pseudobinaural rICF, to better match the shallower relationship associated with rICF of IC neurons (Mc Laughlin et al., 2008, 2014). Unfortunately, rICF data are not available for MSO, but the number of expansive rICFs is similar for IC and superior olivary complex neurons (Coffey et al., 2006). To find the coincidence parameters that would result in physiologically plausible outputs, we started by fitting a power function (Shackleton et al., 2005) to the pseudobinaural rICF,

(2) $$R\left(\rho \right)=a+b{\left(\frac{1+\rho }{2}\right)}^{p}$$

where *R* was the number of coincidences, ρ was the inter-token correlation, and *a*, *b* and *p* were free parameters, but could not be negative. Therefore the curvature of the rICF was completely expressed by the power *p*. As in the previous paragraph, we calculated the quality factor *Q* of the fit, which had to be at least 0.7. Furthermore, the power value had to be between the 10% and 90% quantile of values found earlier for binaural datasets from the cat IC, respectively 0.664 and 4.69 (*N* = 29) (Figure 3B; Mc Laughlin et al., 2008). When the dependence of power *p* on different simulation parameters was studied (Results, Figure 7), this acceptance criterion was ignored, because it would lead to an artificial restriction of values of *p* to the range observed empirically in the IC.

#### Peak rate

Because an adequate number of output coincidences was one of the main concerns in this study (see Introduction), we compared the output rate of our coincidence simulations to that of real binaural recordings. Very few NDFs have been reported for MSO in cat or other species. We used NDFs obtained from axonal recordings of the chinchilla LL for this comparison, presumed to be derived from MSO axons projecting to the IC (Bremen and Joris, 2013). One NDF per fiber was selected. NDFs with a central trough instead of a central peak were discarded, as well as NDFs from fibers with a CF above the maximum tolerated CF for TB and AN fibers (see above). If CF was not available, DF was used instead. The maximal rate of the NDF of the coincidence simulations to correlated sounds had to be between the 10 and 90% quantile of the maximal rate found in this population of LL recordings, i.e., between 19.9 and 158 Hz (*N* = 30) (Figure 3C).

#### Modulation depth

A further important feature of NDFs is the variation in spike rate between peaks and troughs. We expressed this as modulation depth (*MD*),

(3) $$\text{MD }=\frac{\left(\text{peak rate-trough rate}\right)}{\text{peak rate}}$$

where peak rate was the rate at the central peak, and trough rate was the mean of the minimum spike rate on opposite sides of the central peak (Joris, 2003; Smith and Delgutte, 2007). Again this value was compared to that found in the LL recordings. The lower limit of acceptance was the 10% quantile of *MD* for LL NDFs, i.e., 0.749 (Figure 3D; *N* = 30; Bremen and Joris, 2013). There was no upper limit because the maximum *MD* for LL fibers is 1.

#### Halfwidth

The very narrow central peak of the correlograms obtained in the AC analysis of TB fibers (Louage et al., 2005) is strikingly different from the broader central peak of NDFs in MSO or IC neurons (Yin et al., 1986; Yin and Chan, 1990). Convergence of inputs in our coincidence simulations, combined with binaural compression, might increase the width of the main peak because more inputs could generate more coincidences at less favorable ITDs. The halfwidth of the central peak of the NDF was defined as the width of the central peak at the level midway between the peak value and the mean value of the neighboring troughs. Upper and lower limits of acceptance of this measure were defined in the LL dataset (*N* = 28) relative to CF or DF. These limits are shown in Figure 3E as red dashed lines, i.e., resp. −6.01 × 10^−4^ × CF + 1.64 and 8.94 × 10^−5^ ms/Hz × CF + 0.132.

Yin and Chan (1990) show responses to noise for five MSO neurons. We verified that peak rate, modulation depth and halfwidth for these neurons are between the limits derived from the chinchilla data (Dr. T.C.T. Yin, personal communication).

### Statistical analysis

Group data are reported as mean ± standard deviation (SD) unless stated otherwise. Group data between conditions are compared with an unpaired or paired *t*-test as indicated. Statistical significance is defined as a *p*-value ≤ 0.05.

## Results

### Estimation of minimal *N*

**Figure 4:**
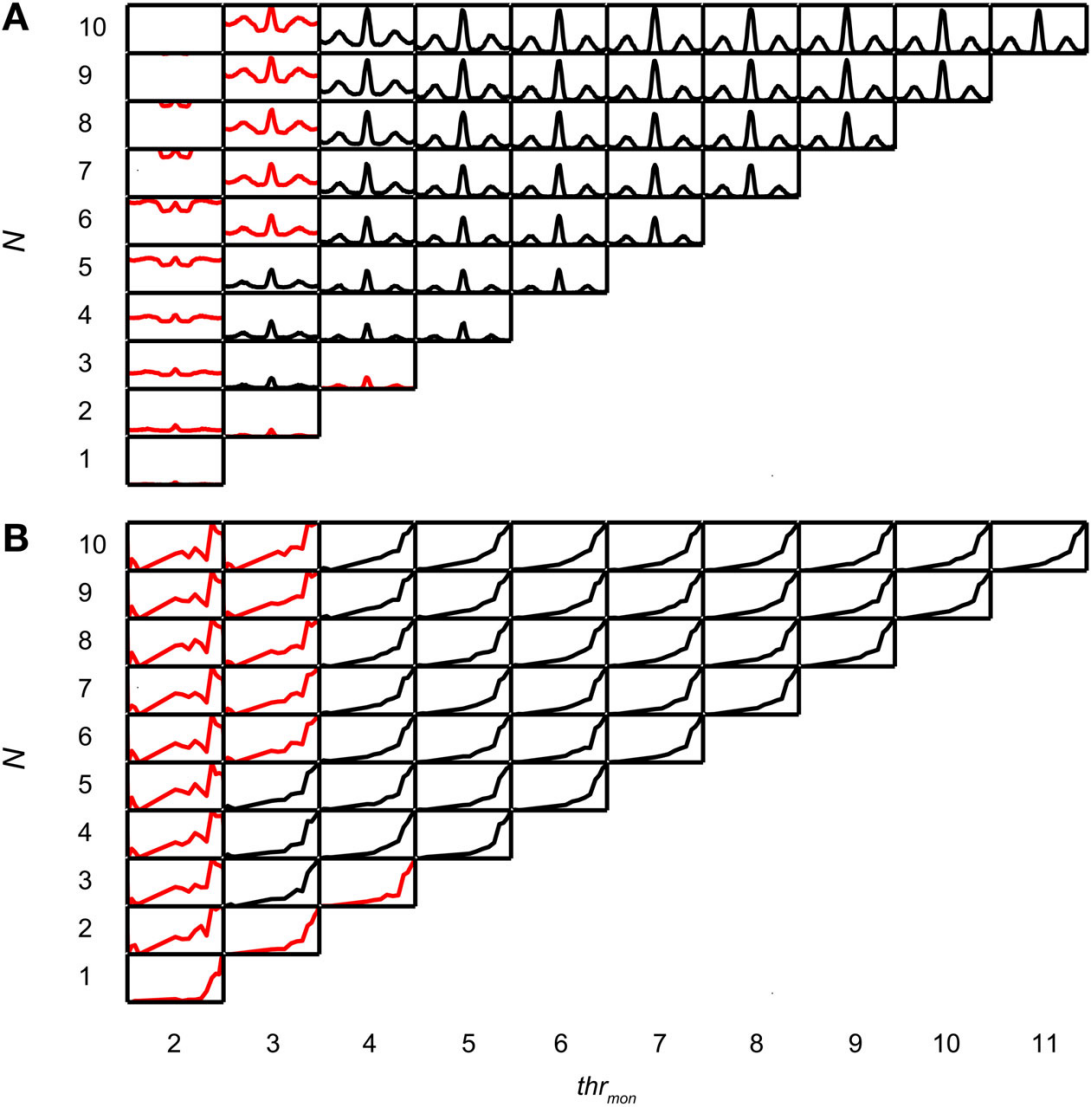
**Simulation output for TB dataset.** *CF* = 559 Hz. *thr_bin_* = 2 and *cw* = 50 μs. **(A)** NDFs to correlated noise, for several values of *N* and *thr_mon_*. For each subplot, delay (abscissa) ranges from −2.94 to 2.94 ms, and coincidence rate (ordinate) ranges from 0 to 170 spikes/s. Black traces indicate accepted simulations, red traces indicate unaccepted simulations (i.e., simulations that fail on at least one acceptance criterion, either for NDF or rICF). **(B)** rICFs for the same dataset and simulation parameters as in **(A)**. For each subplot, the abscissa ranges from ρ = −1 to 1, and the ordinate ranges from 0 to the maximal spike rate of each rICF.

Coincidence rates obtained with the AC analysis are much lower (Louage et al., 2005) than spike rates from actual binaural neurons (Figure 3C). Therefore we first determined the required *N* to get a physiologically plausible output rate. For this analysis, we chose the lowest possible *thr_bin_*, i.e., 2, to maximize the probability of obtaining high output rates. To maximize ITD sensitivity, we chose a low value for *cw*: 50 μs. *N* was varied from 1 to 10 and *thr_mon_* was varied from 2 to 11. Simulation output for these parameters are shown in Figures 4A,B for one TB fiber (*CF* = 559 Hz; spontaneous rate (*SR*) = 1.27 spikes/s). Figure 4A shows NDFs to correlated noise for different *N*, varied in the vertical direction, and for different *thr_mon_*, varied from left to right. The ordinate is from 0 to 170 spikes/sec for all subplots while the abscissa is always from −2.94 to 2.94 ms. For most parameter choices, the NDF shows a clear, damped oscillatory shape. The black traces in Figure 4 correspond to the simulation results that passed all acceptance criteria, while the results in red did not. The maximal value for *thr_mon_* was *N* + 1, because cases with higher *thr_mon_* would be identical (effective monaural coincidences were absent for *thr_mon_* ≥ *N* + 1).

Increase of *N* at a fixed *thr_mon_* (e.g., vertical column at *thr_mon_* of 2, Figure 4A), indeed caused an increase in spike rate, but also a general “DC shift upwards” of the entire NDF with a decrease of modulation depth (*MD*), i.e., a decrease in ITD sensitivity. Such shifted functions are not typically seen in binaural neurons and are caused by an increasing number of monaural coincidences. For higher values of *thr_mon_*, e.g., 6, there is hardly any such upward shift of the NDF with increasing *N*. On the other hand, when increasing *thr_mon_* (i.e., moving horizontally to the right in Figure 4A) for a fixed *N* (e.g., 7), the entire NDF moved down, because of a decreasing number of monaural coincidences for higher values of *thr_mon_*. Note that, beyond a certain value (e.g. 4, for 7 inputs), further increases in *thr_mon_* had no further effect on the NDF, which indicates that it was rare to have that large a number of input spikes from one side coinciding in one *cw*.

Thus, a first conclusion is that increasing *N* can bring a simple coincidence detector to physiological output rates, but this needs to be accompanied by a mechanism to prevent monaural coincidences from being too effective in generating spike output.

Figure 4B shows the corresponding rICFs for the same TB fiber and the same parameter variations. The ordinate is normalized for all subplots from the minimal to the maximal spike rate for that subplot. The abscissa ranges from interaural correlation −1 to +1. The spike rate increases in every subplot with increasing ρ. For a low *thr_mon_*, e.g. 3, higher *N* (e.g., compare 9 inputs to 2 inputs) resulted in a relatively larger spike rate for lower values of interaural correlation. This resulted in an overall less expansive shape of the rICF for higher *N*. This could be due to the “binaural compression” mentioned in the Introduction (but see section Effect of Input Convergence on Expansiveness of rIC Function). Note however that this decrease in expansion of the rICF was rather subtle except for a large change in *N*.

**Figure 5:**
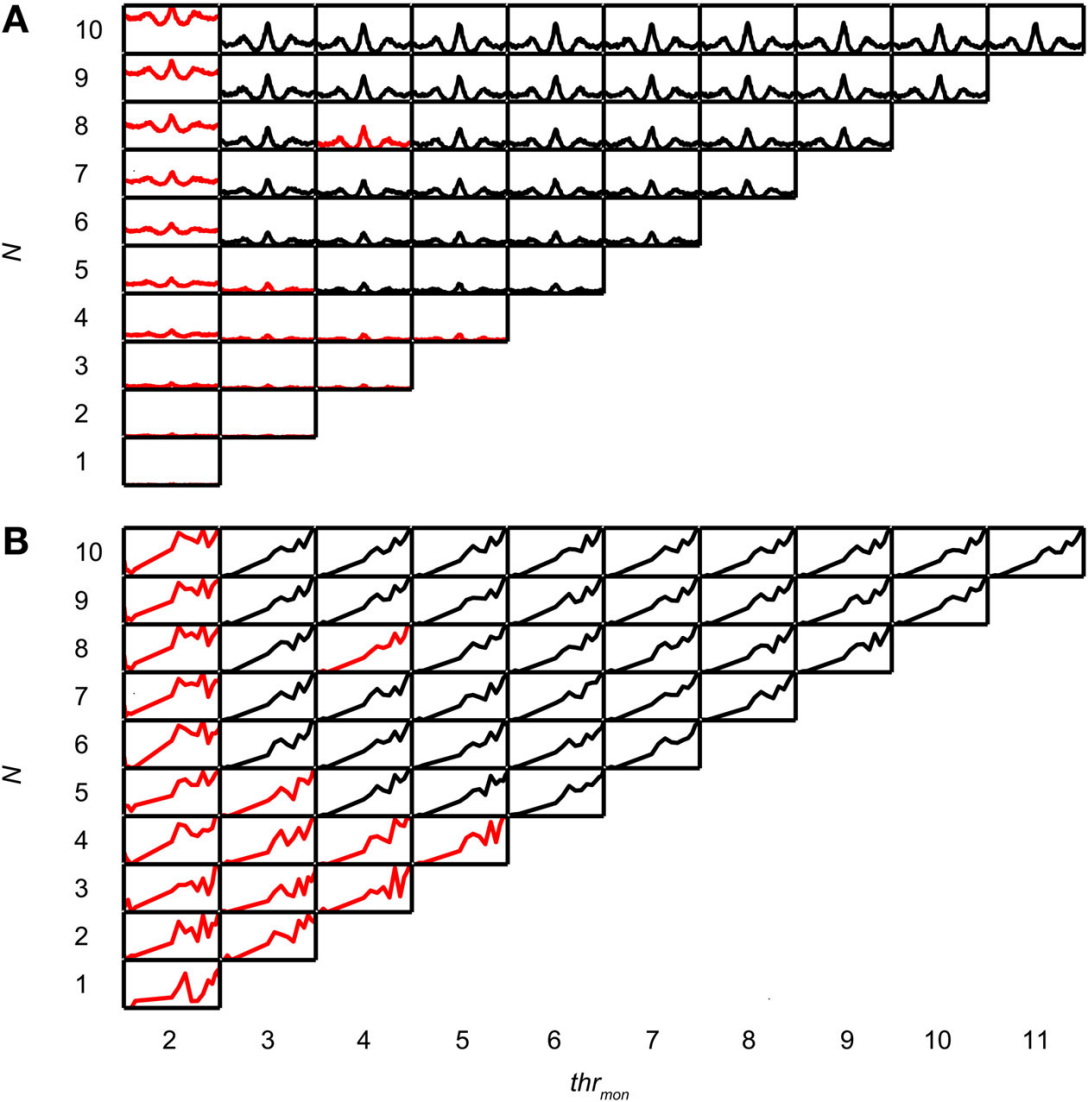
**Simulation output for AN dataset.** *CF* = 544 Hz. *thr_bin_* = 2 and *cw* = 50 μs. **(A)** NDFs to correlated noise of simulations for one AN dataset, for several *N* and *thr_mon_*. For each subplot, delay (abscissa) ranges from −2.94 to 2.94 ms, and coincidence rate (ordinate) ranges from 0 to 125 spikes/s. **(B)** rICFs for the same dataset and simulation parameters as in **(A)**. For each subplot, the abscissa ranges from ρ = −1 to 1, and the ordinate ranges from 0 to the maximal spike rate of each rICF.

Figure 5A shows NDFs for an AN fiber with similar CF (544 Hz, *SR* = 65.2 spikes/s). As in the TB fiber, increase of *N*, while keeping *thr_mon_* low, caused an unphysiological upward shift of the entire NDF (*thr_mon_* = 2). This was easily offset by a slight increase in *thr_mon_* to 3 or higher. The rICF (Figure 5B) was much more linear than for the TB fiber, and this was the case for virtually all parameter combinations, except for the functions for which *thr_mon_* = 2. Maximal spike rates were lower than for the TB fiber, both for the NDFs (note the difference in ordinate between Figures 4A and 5A) and the rICFs (not visible due to normalization). Another difference with the simulation results for the TB fiber is that a somewhat higher number of fibers was necessary to have accepted results (5 for AN, vs. only 3 for the TB).Twelve out of 15 TB datasets (80.0%) and 12 out of 16 AN datasets (75.0%) generated at least one accepted simulation for the chosen simulation parameters. The average required *N* was 3.92 ± 1.62 (mean ± SD) for the TB datasets, and 4.17 ± 0.72 for the AN datasets, but this trend was not significant (two-sample one-tailed *t*-test *p* = 0.685).

**Figure 6:**
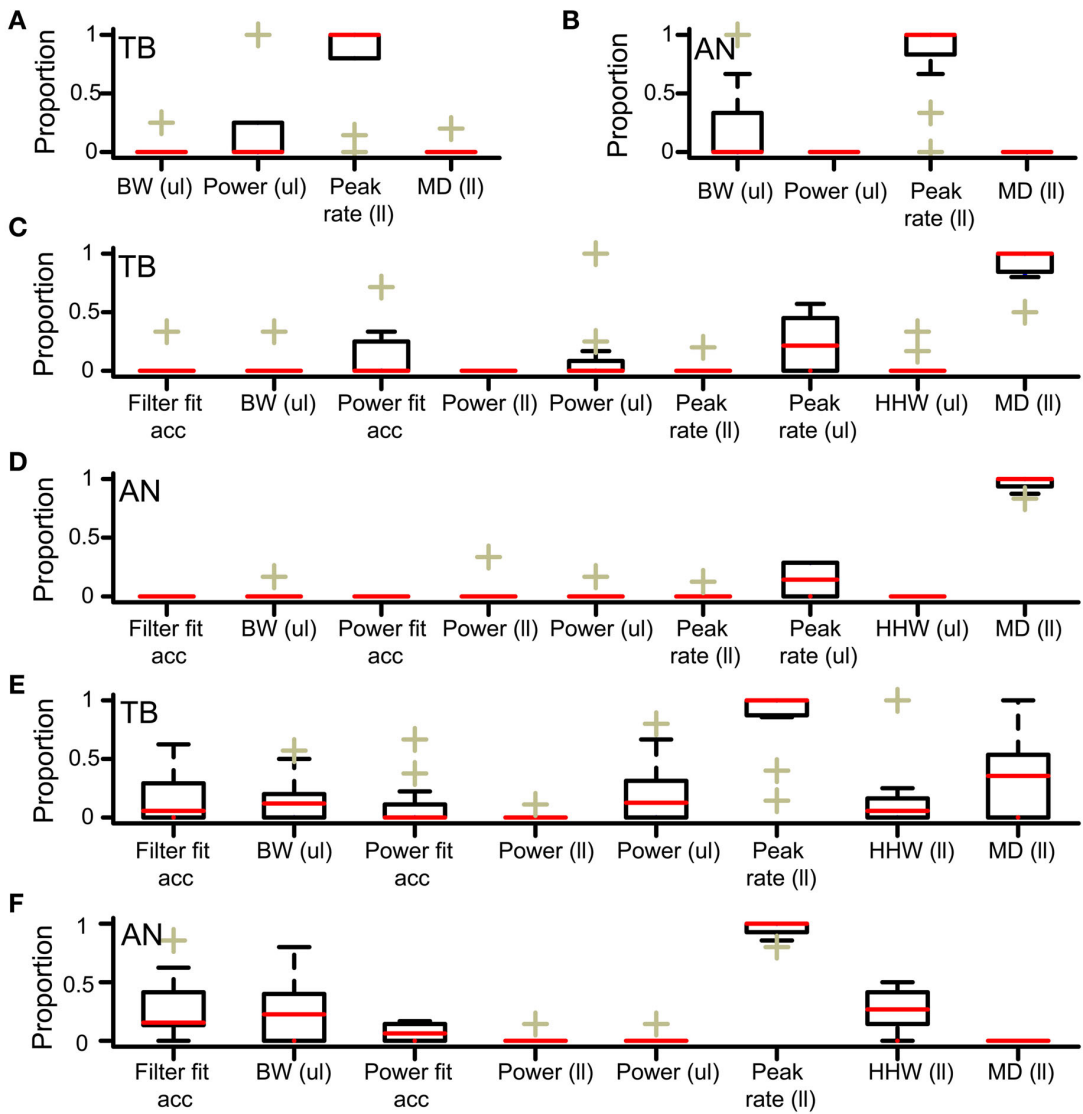
**Acceptance criteria responsible for failure of simulations (A,B).** Acceptance criteria responsible for failure of simulations with one unilateral input less than the minimally required *N*. Only criteria involved in at least one simulation failure are shown. The proportion of failed simulations due to each criterion is shown for all datasets. Each box is bordered by the upper and lower quartile, and the median is indicated by a red line. The whiskers indicate a range of 1.5 times the interquartile range. The plusses indicate values lying beyond this range. **(A)** TB fibers (*n* = 10). **(B)** AN fibers (*n* = 12). ul, upper limit; ll, lower limit. **(C,D)** Criteria responsible for acceptance failure of simulations with *thr_mon_* one lower than the minimally required value. **(C)** TB fibers (*n* = 12). **(D)** AN fibers (*n* = 12). **(E,F)** Criteria responsible for acceptance failure of simulations with *thr_bin_* one higher than the maximally accepted value. **(E)** TB fibers (*n* = 12). **(F)** AN fibers (*n* = 12).

We investigated why low *N* did not result in accepted simulations, for simulations with *thr_bin_* = 2. In this analysis, columns where all cases were rejected (e.g. leftmost column in Figure 4) or accepted (columns for *thr_mon_* > 4 in Figure 4) were not informative and therefore not included. First, we determined the lowest *N* for accepted cases, for all values of *thr_mon_*. For the example in Figure 4, the minimal *N* for *thr_mon_* 3 and 4 are respectively 3 and 4. Then, for each value of *thr_mon_*, the failed case was identified that had just one input less. In Figure 4, these are the cases with *thr_mon_* = 3 and *N* = 2; *thr_mon_* = 4 and *N* = 3. Next, for each of these unaccepted cases, we listed the failed criteria. For the example in Figure 4, the case with *thr_mon_* = 3 and *N* = 2 failed because the *p* of the associated rICF was too high. The case with *thr_mon_* = 4 and *N* = 3 failed for the same criterion. This information was then summarized per dataset by dividing the number of times a particular acceptance criterion failed, by the number of failed cases. For the example in Figure 4, this resulted in 1 (2 times divided by 2 cases) for the acceptance criterion “upper limit of power.” None of the other acceptance criteria failed in these cases and thus score 0. This procedure was performed for each TB dataset, and pooled and summarized for all datasets as boxplots in Figure 6A. The most frequent reason why simulations with low *N* were not accepted is that they resulted in a NDF peak spike rate that was too low. This was also the case for the AN (Figure 6B).

In summary, for *cw* = 50 μs and *thr_bin_* = 2, a minimum of 4 input fibers from each side was typically necessary, primarily to achieve physiologically plausible output rates. To obtain physiologically plausible output for higher *N*, it was critical that monaural coincidences are suppressed (see section Possible Values of *thr_mon_*).

### Effect of input convergence on expansiveness of RIC function

**Figure 7:**
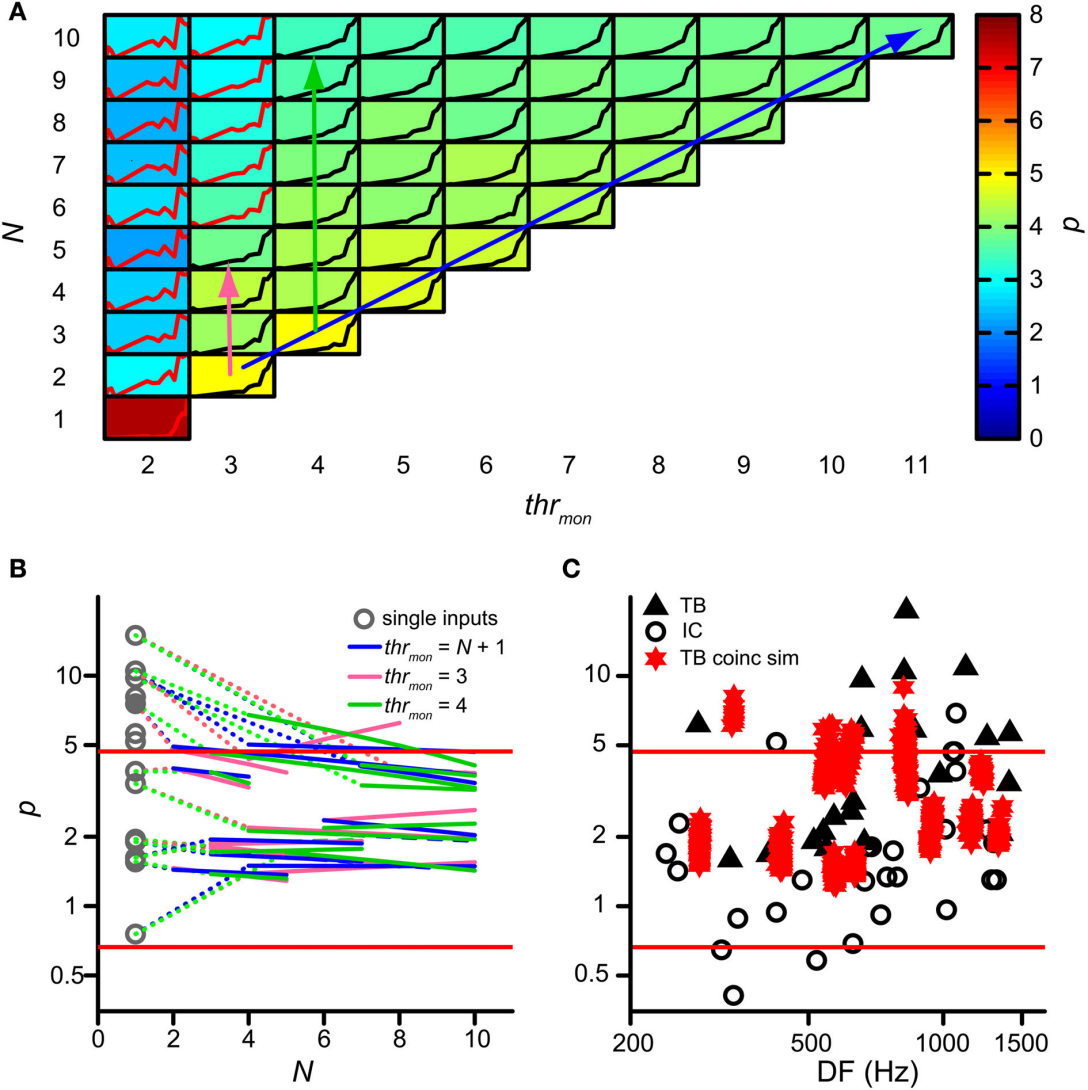
**Effect of convergence of inputs on *p* of rICF. (A)** Set of simulations for one TB dataset (same as in Figure 4). *N* and *thr_mon_* are varied; *thr_bin_* = 2 and *cw* = 50 μs. In each subplot the rICF corresponding to that combination of simulation parameters is shown. Black traces represent accepted simulation results. Subplot background color represents the power value. The abscissa in each plot ranges from −1 to 1; the ordinate ranges from 0 to the maximum of the particular rICF. **(B)** TB population data showing change of power with convergence of inputs. Gray circles represent the power for the simulations corresponding to the single input correlograms. Colored lines are fit through accepted simulations, where different colors correspond to the arrows in **(A)**. Dotted lines connect the power of the single input correlogram to these fits. **(C)** Comparison of power *p* of rICFs: IC data (circles) are repeated from Figure 3B. TB data (triangles) taken from (Mc Laughlin et al., 2014). Values of *p* for otherwise accepted simulations (red stars) are added for 13 TB datasets. Solid red lines represent limits of accepted power values (Figure 3B).

Next we investigated whether binaural compression could lower the power *p* of the rICF. This power is indeed too high in AC analyses of TB fibers where coincidences are counted for a single input from each ear (triangles on Figure 7C; Mc Laughlin et al., 2008, 2014). For the analysis in this section, *p* was removed from the acceptance criteria. Figure 7A shows the value of *p* (background color of each subpanel) for multiple simulations of the same TB dataset as in Figure 4, with varying *thr_mon_* (different columns) and *N* (different rows). Again, *thr_bin_* = 2 and *cw* = 50 μs. Note that single inputs (lower left) resulted in a very high *p* value of ~7.4 (brownish color), consistent with the earlier AC analysis. In that case there are basically no coincidences at all for ρ < 0.5. Increase of *N*, keeping the same *thr_mon_* (=2, leftmost column), caused a precipitous drop to *p* < 3: this is due to coincidences that now occur at ρ < 0.5. A similar but much more gradual tendency was present at other values for *thr_mon_*: the value of *p* shows a gentle decrease with increasing *N*—from yellow to greenish colors. The reverse—a gentle *in*crease in *p* with increase in *thr_mon_*—is generally present within each row corresponding to a fixed *N*. There was a large increase in *p* for an increase in *thr_mon_* from 2 to 4, but effects of further increases in that threshold were modest.

Figure 7B shows the change in *p* for the entire group of TB datasets. Gray circles indicate *p* for simulations with just one input on each side, i.e. the single input correlogram for each dataset. The resulting *p* values could be very high and were often outside the boundaries (red lines) observed in binaural neurons of the IC. This is consistent with the very expansive rICFs reported previously (Mc Laughlin et al., 2008, 2014). Magenta, green and blue lines are fitted through accepted simulations for the parameter combinations indicated by the respective colored arrows in Figure 7A: the diagonal arrow corresponds to simulations without effective monaural coincidences, because *thr_mon_* is always higher than *N*; for the vertical arrows, *thr_mon_* is fixed, and the number of monaural coincidences increases with *N*. Clearly, the largest effect on *p* occurred by increasing *N* from 1 to >1, and this effect was larger when *p* for *N* = 1 was very high. Overall there was a slight further power decrease with increasing *N*. The average slope of the simulations without monaural coincidences (blue lines; mean −0.075; one-sample one-tailed *t*-test *p* = 0.001) and of the simulations with *thr_mon_* = 4 (green lines; mean −0.11; one-sample one-tailed *t*-test *p* = 0.016) were statistically significantly lower than 0, whereas the average slope of the simulations with *thr_mon_* = 3 (magenta lines; mean −0.031; one-sample one-tailed *t*-test *p* = 0.32) was not. Therefore, having more than one input on each side could dramatically decrease the power, but the additional effects of increases of *N* > 2 were very small.

For a more direct comparison, Figure 7C shows the range of powers for the accepted model simulations and compares them with the values of IC neurons (circles) and the single input correlograms of TB fibers (solid triangles). The powers corresponding to the accepted simulations of TB fibers (red stars) were generally below the high values previously reported for the AC analysis (Mc Laughlin et al., 2014) and closer to those of binaural IC neurons, but the mean value was still higher than for the IC dataset (mean ± *SD*, *N* for simulations: 3.12 ± 1.65, 13 vs. IC: 2.05 ± 1.58, 29, one-tailed *t*-test, *p* = 0.027). Thus, convergence of inputs capped the power to values within the range of experimental binaural recordings, but they were still in the upper part of that distribution.

**Figure 8:**
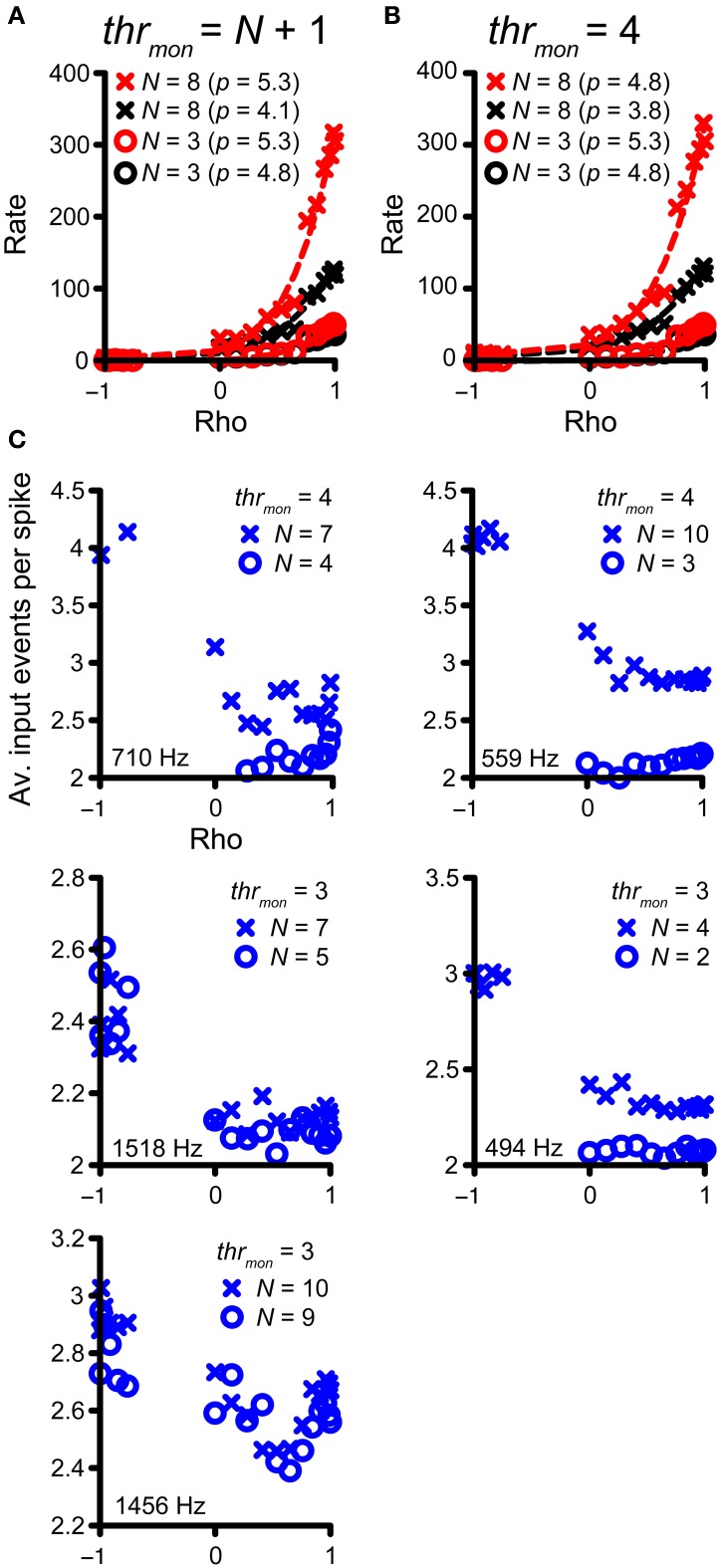
**Compression of rICF with convergence of inputs. (A,B)** The same TB dataset as in Figure 4 is shown. In all cases, *thr_bin_* = 2 and *cw* = 50 **μ** s. rICFs are for simulations with *N* = 3 (circles) and *N* = 8 (crosses). **(A)** Simulations for output without monaural coincidences. *thr_mon_* is 4 and 9, respectively. The power *p* of the fit is stated in the caption. **(B)** rICFs with the same number of inputs as in **(A)**, but now the *thr_mon_* is fixed at 4. The ordinate in **(B)** is the same as in **(A)**. Dashed lines show the fits. **(C)** The average number of input events per successful output spike, as a function of ρ. Five TB datasets were selected that show the largest decrease in *p* with *N*, for *thr_mon_* = 3 or 4. Crosses correspond to the high *N* (lowest *p*), circles correspond to the low *N* (highest *p*). CF is indicated in each panel.

Previously, in Figure 4, we highlighted the increase in number of monaural coincidences with increase in *N* (thus along the magenta and green vertical arrows in Figure 7A). However, the occurrence of monaural coincidences was not required for a decrease of rICF expansiveness with a higher *N*, because it could also be seen for conditions without monaural coincidences (blue arrow in Figure 7A and blue lines in Figure 7B). Example rICFs are shown in Figure 8A for a TB fiber dataset (*CF* = 559 Hz): black circles correspond to 3 and black crosses correspond to 8 inputs, respectively. In both cases, *thr_mon_* > *N*, so there were no effective monaural coincidences. The power (indicated in the caption) is lower for higher *N*: 4.1 for 8 inputs vs. 4.8 for 3 inputs. More inputs means a larger probability of having a (binaural) coincidence: having more inputs at both sides increases the chance that any two of them will fire at the same time, because the number of combinations of 2 out of *N* inputs increases with *N*. This effect is larger if there are not yet that many coincidences, i.e., for suboptimal values of ρ, but diminishes when there are many coincidences because there can only be one output spike per *cw*. Thus the lowering in *p* here could reflect the “binaural compression” mentioned in the Introduction. The red traces correspond to the black traces, but without application of the refractory period. Now there is no decrease in *p* anymore for the higher number of inputs. This suggests that the main reason for a lower *p* is not binaural compression, but rather spike rate saturation due to the refractory period. In Figure 8B, rICFs are shown for a fixed *thr_mon_* = 4. This threshold enabled monaural coincidences for a high *N* (8) but not for a low *N* (3). The effect is a lowering of *p* for *N* = 8 (from 4.8 to 3.8). Now there is also a lowering of *p* without the refractory period (from 5.3 to 4.8, with increase in N from 3 to 8), in which perhaps “binaural compression” plays a role.

To explore this possibility more directly, we selected the five TB fiber datasets with the largest decrease in *p* for increasing *N*, for either *thr_mon_* = 3 or 4. The accepted rICFs with the lowest and highest *p* were identified and are analyzed in Figure 8C. Here, the ordinate shows the average number of coinciding input spikes per output spike (for the non-refracted spikes, thus corresponding to the red functions in Figure 8B), and this average is shown as a function of ρ. The prediction is that “binaural compression” is highest for high ρ and for the largest *N*. We find that for negative ρ, the average ratio of input to output spikes is close to *thr_mon_*. This is not surprising: at these ρ values, the vast majority of coincidences are monaural, and therefore the ratio is close to *thr_mon_* (3 or 4). For ρ > 0, the ratio of input to output spikes is lower than for ρ < 0, reflecting the increasing number of binaural coincidences, for which the threshold is lower (*trh_bin_* = 2). If “binaural compression” would be responsible for the lower *p* in case of high *N*, the average number of input events per spike would increase toward ρ = 1, and more so for high *N* than low *N*. However, it can be seen that this pattern is not consistently present. Instead, the average number of coinciding events is low, and does not markedly increase toward ρ = 1. We conclude that there are usually too few coinciding input events to get significant “binaural compression.”

To summarize, the convergence of multiple inputs softened the extreme expansiveness seen in the single-input rICF of many TB fibers but the influence was minor beyond a convergence ratio of 2 fibers per side. The main factor appears to be refractoriness (i.e., a ceiling on firing rate per cycle) rather than “binaural compression” (i.e., the sublinear relation between *N* and output spike rate that is inherent to the coincidence mechanism).

### Possible values of *thr_mon_*

**Figure 9:**
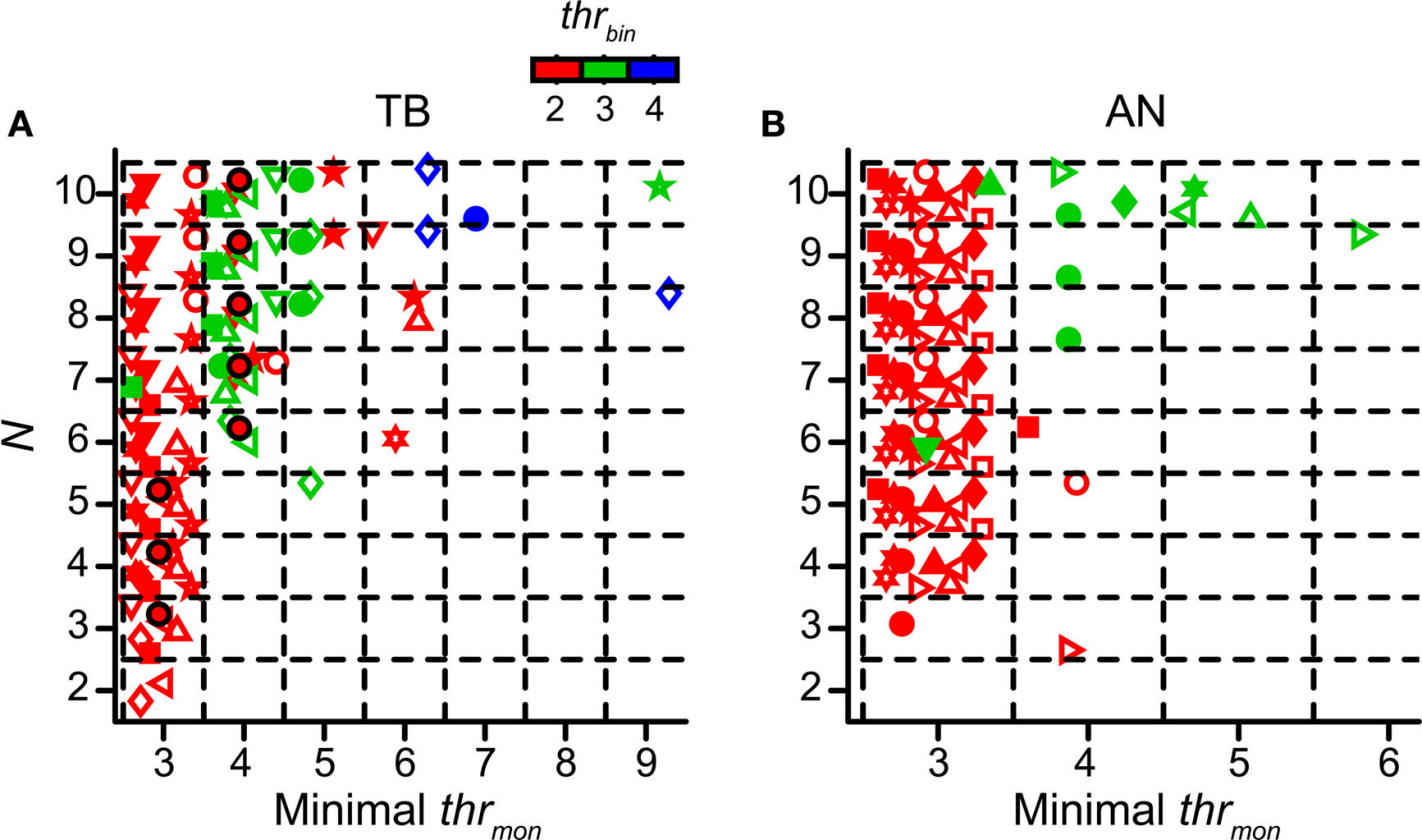
**Minimal values of *thr_mon_* of accepted simulation cases.** Simulations with TB **(A)** (*n* = 12) and AN **(B)** (*n* = 13) datasets. Different colors represent different values of *thr_bin_*. Each symbol represents the minimal *thr_mon_* values for the accepted cases of one dataset. Black circles filled with red represent dataset in Figure 4. Symbols are jittered to decrease overlap. *cw* = 50 μs.

We already mentioned the fact that a higher *thr_mon_* helped to prevent an unphysiological upward shift of the NDF for more inputs (Figure 4). In this section we explore the values of *thr_mon_* for accepted simulations. Again *cw* was set at 50 μs. The other simulation parameters were varied. As was the case for the TB simulations shown in Figure 4, there was typically a minimal *thr_mon_* associated with acceptable simulations. For the fiber of Figure 4 the minimal *thr_mon_* was 3 for *N* = 3, 4, or 5, and 4 for higher *N*, in each case keeping *thr_bin_* = 2. The layout of Figure 9A is similar to that of Figure 4 but now the abscissa shows the minimal *thr_mon_* of accepted simulations for TB datasets. Different datasets are indicated with different symbols, and different colors indicate different values for *thr_bin_*. So, for example, the data shown in Figure 4 are for a *thr_bin_* of 2 and are shown in Figure 9A as the black circles filled with red. These are the most leftward accepted (black) combinations in Figure 4. Figure 9B shows the same data for AN datasets.

The general message from Figures 9A,B is that *thr_mon_* almost invariably needed to be higher than *thr_bin_* in order to obtain acceptable simulations. Across all simulations for *thr_bin_* = 2 (red symbols), we found that the mean minimal value of the *thr_mon_* for accepted simulations was 3.08 ± 0.29 for TB fibers and 3 ± 0 for AN fibers. For *thr_bin_* = 3 (green symbols), the mean of the minimal *thr_mon_* increased to 4.5 ± 1.85 for TB, and 4.13 ± 0.83 for AN. When the minimal *thr_mon_* was divided by *thr_bin_*, we obtained a mean of 1.42 ± 0.23 for TB datasets and 1.38 ± 0.18 for AN datasets. For *thr_bin_* = 4 (blue symbols), there were few accepted simulations (none for AN), even for the maximal number of inputs tested (*N* = 10).

Because the number of monaural coincidences increases with *N* (Figure 4), it is expected that the minimal *thr_mon_* will rise with *N* (Figure 9). It is therefore useful to express the minimum *thr_mon_* as a fraction of *N*. This average was 0.42 ± 0.14 for TB and 0.30 ± 0.0096 for AN fibers (two sample *t*-test p = 0.0061; *thr_bin_* = 2). This means that the requirement of *thr_mon_* was more stringent for TB fibers than for AN fibers. Indeed, in the example in Figure 5 (*thr_bin_* = 2), the simulations for *thr_mon_* = 3 were acceptable for 6 inputs or more: for lower *N*, the output spike rate was too low. In contrast, the same parameters for the TB fibers of Figure 4 gave rise to a “DC problem” once *N* exceeded 5. This suggested that the enhancement of temporal coding between AN and TB (Joris et al., 1994a,b; Louage et al., 2005) comes at the cost of increasing the probability of effective monaural coincidences, unless this enhancement is accompanied by an increase of *thr_mon_* (relative to *thr_bin_*).

The boxplots in Figures 6C,D show the acceptance criteria determining the minimal *thr_mon_* for simulations with *thr_bin_* = 2. The most important criterion was the lower limit of the *MD*, both for TB datasets (panel C, *N* = 12 datasets) and for AN datasets (panel D, *N* = 12 datasets). This again indicated that a high *thr_mon_* (relative to *thr_bin_*) was necessary for physiological ITD sensitivity *via* suppression of monaural coincidences.

As mentioned in the **Introduction**, the sensitivity of a neuron to binaural coincidences vs. monaural coincidences has been addressed before. Colburn et al. (1990) proposed that the mere phenomenon of convergence would be an explanation for the fact that binaural coincidences are promoted relative to monaural coincidences, because the number of combinations of inputs that could possibly generate a binaural coincidence is higher than that of monaural inputs. The number of combinations *NC_b_* that results in *x* inputs spiking at the same time in the binaural situation with *N* inputs on each side is given by

(4) $$NC_{b}\;=\;\frac{(2N)!}{((2N-x)!\;x!)}$$

The number of combinations *NC_m_* of having *x* monaural inputs spiking at the same time is given by twice the number of combinations for each side separately, or

(5) $$NC_{m}\;=\;2\;\frac{N!}{((N-x)!\;x!)}$$

**Figure 10:**
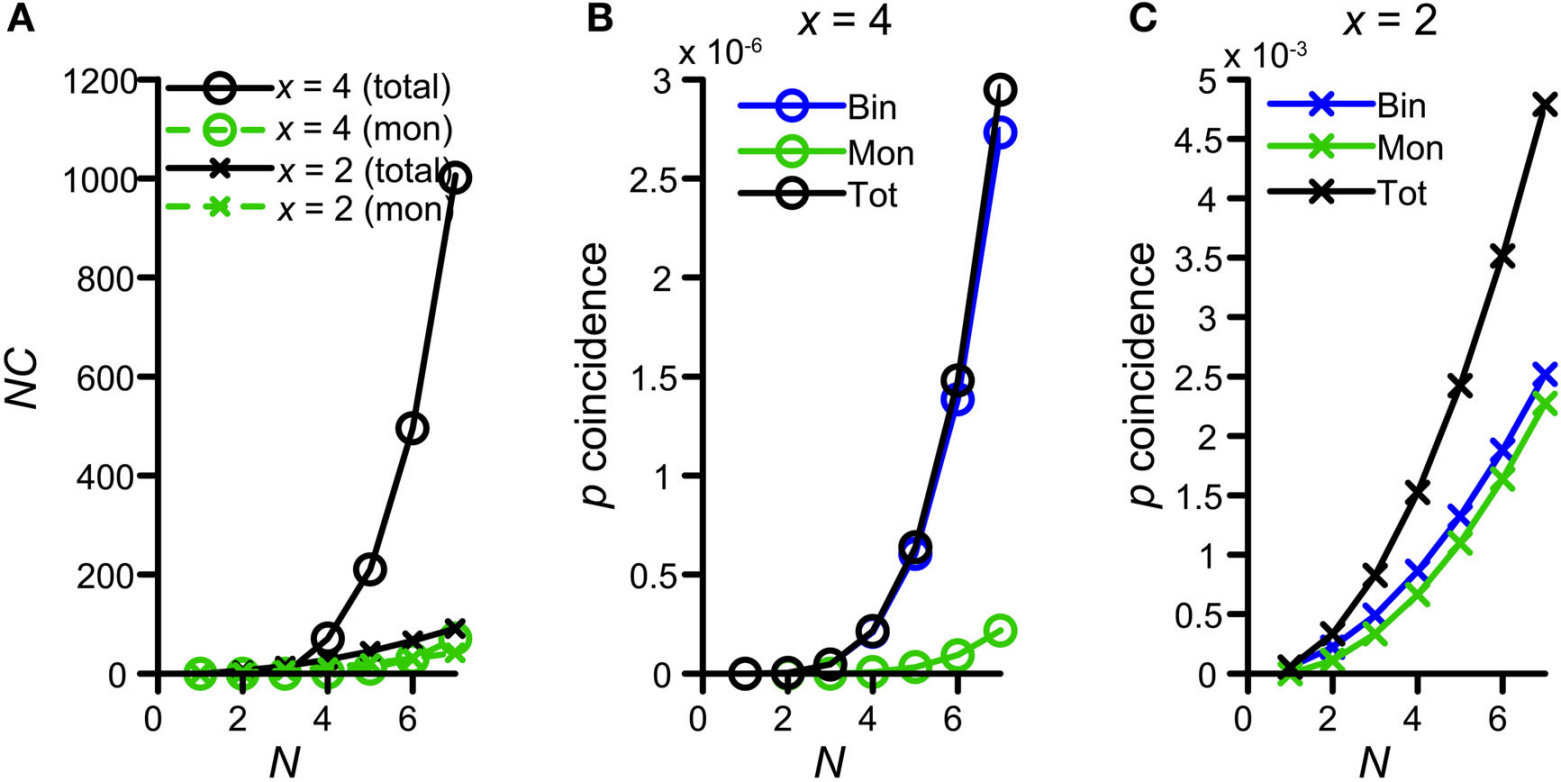
**Graphical display of the argument of Colburn et al. (1990).** See text for detailed explanation. **(A)** Number of combinations for either 4 (circles) or 2 (crosses) coincidences, as a function of *N*. Green traces indicate the possible number combinations monaurally, i.e., twice the number of combinations of either *x* = 2 or 4 out of *N*. Black traces indicate the total (monaural + binaural) possible number of combinations, i.e., either 2 or 4 out of 2*N*. **(B)** Probability of having exactly 4 coincidences monaurally (green trace), binaurally (blue trace) or in total (black trace), as a function of *N*. The chance of having an event on 1 input is *p_s_* = 0.0075. **(C)** Probability of having exactly 2 coincidences monaurally (green trace), binaurally (blue trace) or in total (black trace), as a function of *N*.

Figure 10A shows the number of possible combinations in the binaural and monaural situation, *NC_b_* (black circles) and *NC_m_* (green circles), resulting in 4 simultaneous spikes (*x* = 4), for different numbers of input *N*. For example, if each side receives 5 inputs (*N* = 5) and 4 spikes are required to exceed threshold, there are 210 possible binaural combinations to achieve this but only 10 possible monaural combinations. Because *NC_b_* = 210 for *N* = 5 whereas *NC_m_* is only 10, Colburn et al. (1990) concluded that just this difference in the number of possible permutations might explain the larger sensitivity of MSO neurons for binaural over monaural coincidences. Indeed this translates into a coincidence probability that rises much faster for the binaural situation than for the monaural situation: Figure 10B shows the probability of having 4 monaural coincidences in one coincidence window (green circles), as a function of *N*, assuming that the chance of having 1 event on 1 input is *p_s_* = 0.0075 (corresponding to a spike rate of 150 Hz if *cw* = 50 μs). The blue circles indicate this probability for strictly binaural situations (where every side contributes at least one event), and the black circles show the total binaural probability of having 4 coincidences (adding the strictly binaural and the strictly monaural probabilities). Clearly, the probability that output spikes are driven by binaural coincidences is much higher than that of monaural coincidences.

This theoretical consideration contrasts with our finding that *thr_mon_* needed to be higher than *thr_bin_* (Figures 4,5). This means that the combinatorial effect pointed out by Colburn et al. (1990) does not suffice for all parameter conditions to promote binaural coincidences over monaural coincidences. The discrepancy is explained by the fact that this combinatorial binaural advantage is very dependent on the number of events required to coincide (*x*). Figure 10A (crosses) shows the number of combinations for *x* = 2 coinciding input events. Now the difference between *NC_b_* (black) and *NC_m_* (green) is much smaller, simply because the number of combinations of 2 out of 2*N* grows not that much faster with *N* than twice the number of 2 out of *N*. For example, for *x* = 2 and *N* = 5, *NC_b_* = 45, and *NC_m_* = 20. This leads to a coincidence probability that is not that much larger binaurally relative to monaurally (Figure 10C). Clearly, the probability of having 2 monaural coincident spikes (green) is almost the same as that of having a binaural coincidence (blue).

Of course, for a coincidence detector with *thr_bin_* = 2, there will be some epochs where the effective number of coinciding input events is >2, and for which binaural coincidences are more likely than monaural ones. Note, however, that the absolute probability of having *x* = 4 coinciding events is several orders of magnitude smaller than the absolute probability of having *x* = 2 coinciding events (compare ordinate of panel B vs. panel C in Figure 10), so that such epochs are a small fraction of the total number of epochs that generates an output spike. In summary, requiring a relative large number of coincident spikes (e.g., *x* = 4) has the advantage of automatically favoring binaural over monaural coincidences, but has the drawback of yielding a low probability of coincidences. We explore this further below (section Maximal Value of *thr_bin_*).

If *thr_mon_* > *N* (i.e., the diagonal in Figures 4, 5, 7A), monaural coincidences are effectively removed. Such simulations are shown on the diagonal of these figures (*thr_mon_* = *N* + 1). They illustrate that these parameter combinations can result in physiologically plausible input. One may argue that a complete absence of monaural coincidences is a requirement for an “ideal” binaural coincidence detector.

In summary, for physiological plausible output, it is important to have a *thr_mon_* that is not too low, because monaural coincidences strongly decrease binaural sensitivity. This lower limit increased with *N*. Interestingly, *thr_mon_* was especially stringent for TB fibers, because the high synchronization in their spike trains made the number of monaural coincidences rise fast with increasing *N*.

### Maximal value of *thr_bin_*

**Figure 11:**
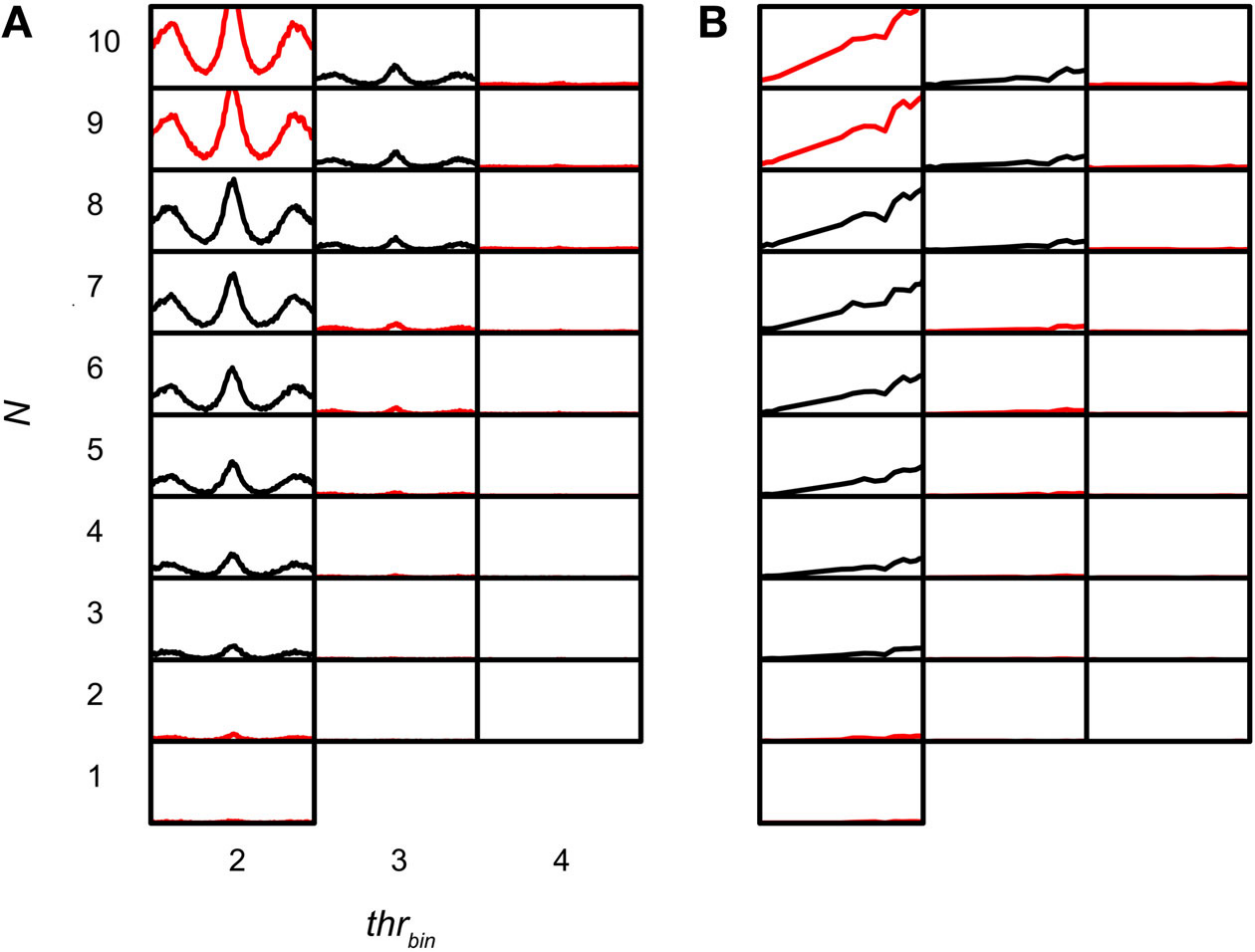
**TB simulation output for several *N* and *thr_bin._* CF 456 Hz; SR 89.9 Hz; *thr_mon_* = 4; *cw* = 50 μs. (A)** NDFs. The abscissa in each subplot ranges from −2.94 to 2.94 ms. The ordinate in each subplot ranges from 0 to 150 Hz. **(B)** rICFs. The abscissa in each subplot ranges from −1 to 1. The ordinate in each subplot ranges from 0 to 150 Hz. *N* and *thr_bin_* are the same in **(A)** and **(B)**.

The sensitivity of the coincidence detector to input events was studied by varying *thr_bin_*. Figures 11A,B shows simulations for which *thr_bin_* and *N* were varied for one TB dataset (CF 456 Hz; SR 89.9Hz; *thr_mon_* = 4). The simulation output decreased drastically when *thr_bin_* was increased above 2. Functions for *thr_bin_* > 4 are not shown because they are almost completely flat at 0 spikes/s. Note that there were two conflicting demands at work, with little margin. If the *thr_bin_* was minimal (2), there was a steady increase in output rate with increasing *N*, as expected (Figure 11, leftmost column). For the highest *N* tested (9 and 10) the output was not accepted because the spike rate at the peak was too high. Increasing the *thr_bin_* just by one gave the opposite result: except for one parameter value (*N* = 8), the accepted simulations of the leftmost column were now unacceptable and *vice versa*. In this case, the problem was the reverse one: the output rate was too low. This suggests that, given the firing rates and temporal characteristics of real TB fibers, and the low number of input fibers per side (see Discussion), permissible values of *thr_bin_* are severely restricted.

**Figure 12:**
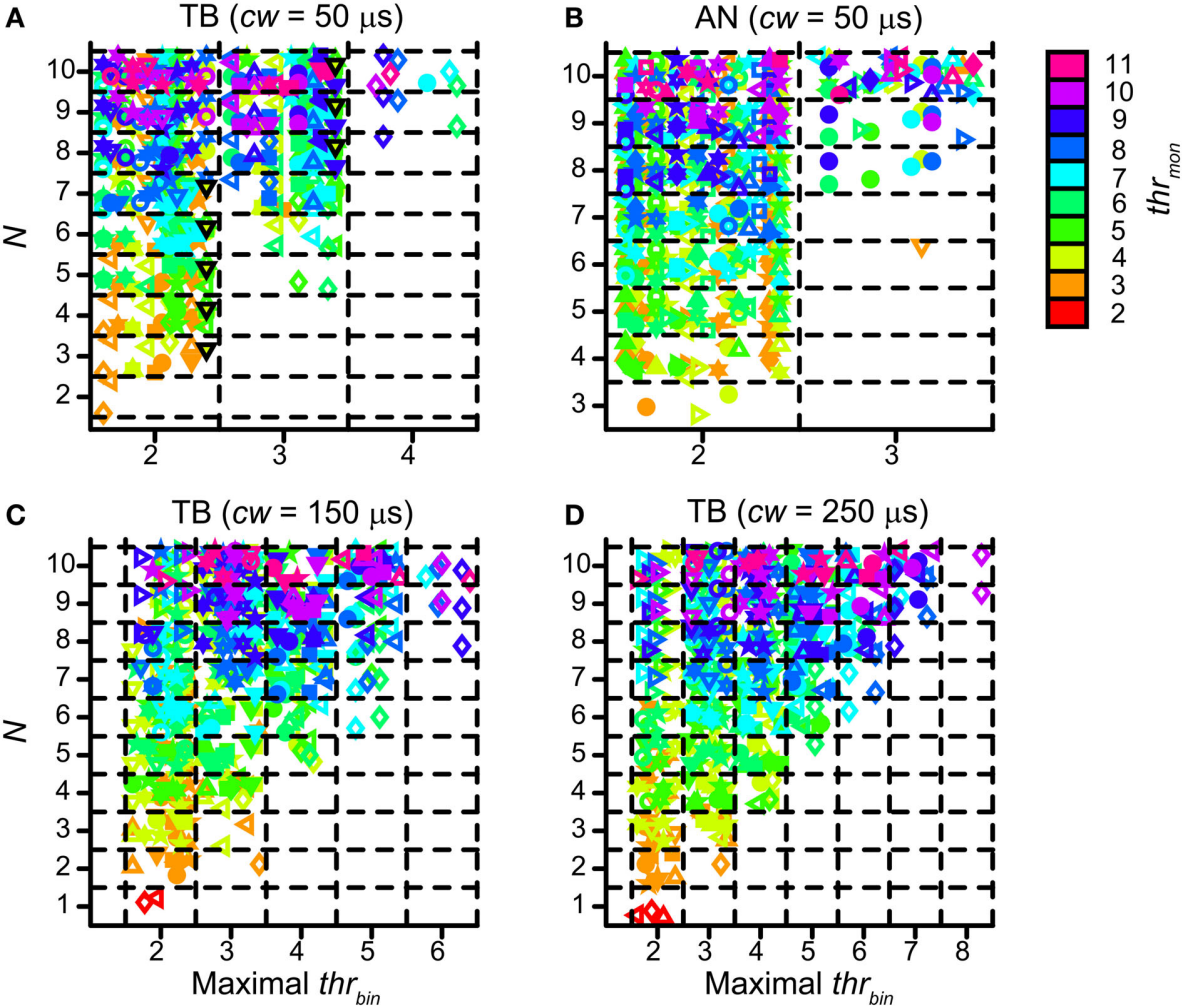
**Maximal values for *thr_bin_*, for different values of *N* and *thr_mon_*. (A)** TB fibers (*n* = 12), *cw* = 50 μs. Black triangles filled with green indicate dataset in Figure 11. **(B)** AN fibers (*n* = 13), *cw* = 50 μs. **(C)** TB fibers (*n* = 13), *cw* = 150 μs. **(D)** TB fibers (*n* = 13), *cw* = 250 μs. Symbols are jittered to decrease overlap.

Population data are shown in Figure 12 for all TB (A) and AN (B) datasets, with *cw* = 50 μs. Each symbol marks the maximal possible *thr_bin_* (indicated on the abscissa) for a particular *N* (ordinate) and *thr_mon_* (color of symbols). As in Figure 9A, different symbols indicate different datasets. The dataset shown in Figure 11A is shown in Figure 12A by black triangles filled with green. For the population, possible values for *thr_bin_* were strikingly limited to only 2, 3 or sometimes 4, both for TB and AN. Thus, even for 10 perfectly matched (in terms of CF, properties, delay) input fibers from each side, a requirement of 3 binaurally coinciding input spikes, i.e., two from one side and one from the other, was practically the maximum threshold that can be imposed. With further increases in *N* (>10, not tested here), it is likely that a higher maximal *thr_bin_* will generate acceptable simulations. Note however that the increase in *N* needed to be accompanied by a sizeable increase in *thr_mon_* (colors).

The boxplots in Figure 6E (TB) and Figure 6F (AN) show which acceptance criteria failed for simulations for which *thr_bin_* was just one step higher than the highest value for accepted simulations. The main limiting factor was the minimal value of the peak rate.

Thus, for the range of simulation parameters explored here, the coincidence detector has to be sensitive to very few (2–3) coinciding input spikes: if too many input spikes were required, the output spike rate was too low.

### Effect of changing *cw*

**Figure 13:**
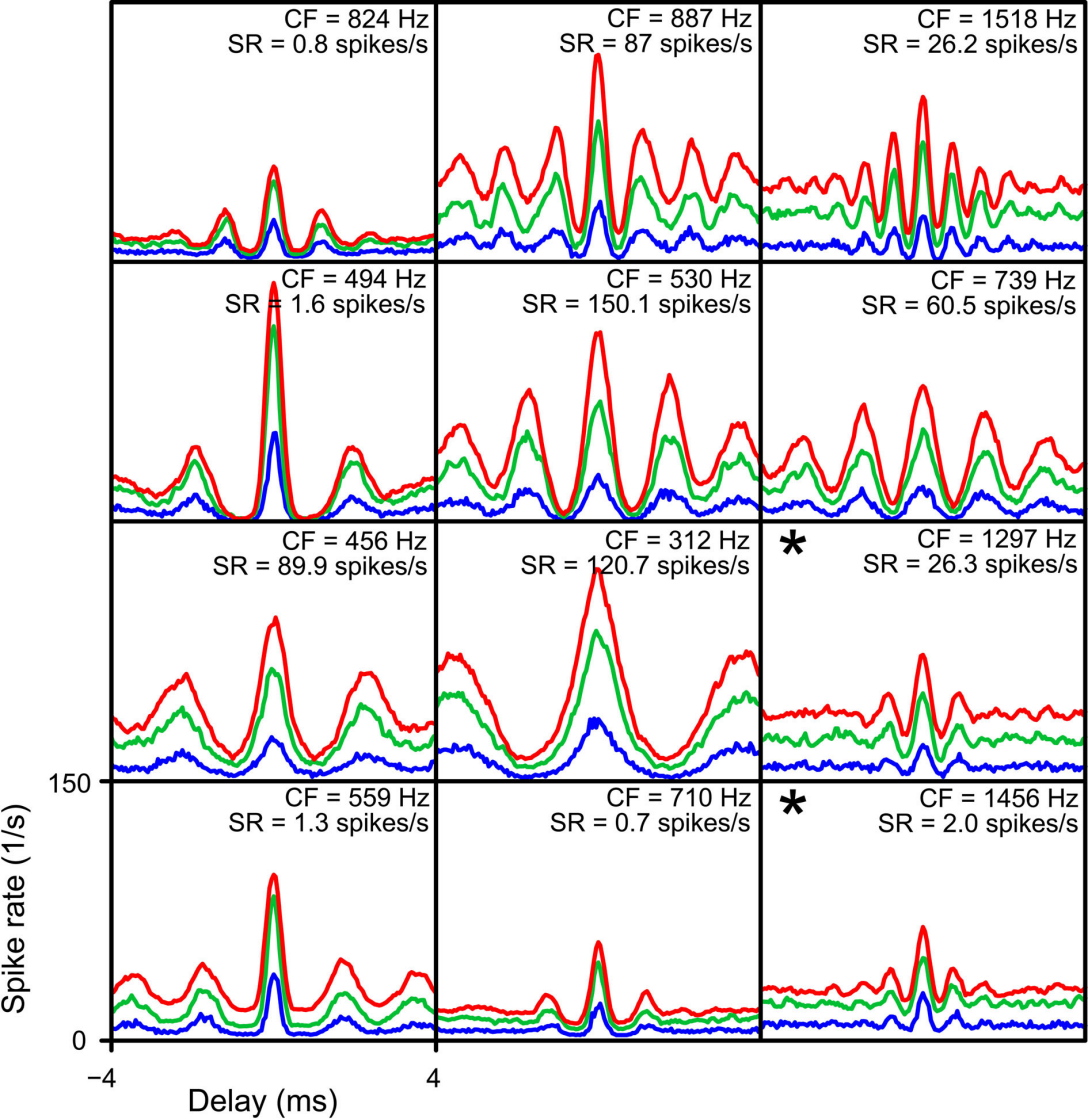
**Simulated NDFs for TB datasets, for different values of *cw*.** In every panel NDFs are shown for one TB dataset, for *cw* = 50 μs (blue), *cw* = 150 μs (green) and *cw* = 250 μs (red). CF and SR are indicated for each dataset. Range of abscissa and ordinate indicated in lower left panel apply to all panels. *thr_bin_* = 2. *N* and *thr_mon_* for each TB dataset are the minimal values to get an acceptable simulation for *cw* = 50 μs. Asterisks indicate simulations where the NDFs for *cw* = 150 μs and *cw* = 250 μs were not accepted.

The final parameter explored is *cw*. Besides the standard value of 50 μs, we evaluated coincidence simulations with *cw* = 150 and 250 μs. In Figure 13 NDFs are shown for TB datasets for these different *cw*. Again, *thr_bin_* was fixed at 2. For every dataset, the combination of lowest *N* and *thr_mon_* was chosen that resulted in accepted simulations for a *cw* of 50 μs (corresponding to the blue NDFs). The abscissa ranges from −4 to 4 ms, and the ordinate from 0 to 150 Hz. These parameters were then kept the same for simulations with *cw* 150 (green) and 250 (red) μs, respectively. It can be seen that an increase of *cw* led to a general increase in spike rate, without a drastic change in shape of the functions. However, for many datasets the increase in spike rate was accompanied by a decrease in *MD*, again due to an upward “DC-shift.” The larger *cw* resulted in accepted simulations for 10 out of 12 TB datasets. In the remaining 2 datasets (indicated with asterisks), the cause of rejection of the simulation was a *MD* below the lower limit (both for *cw* = 150 μs as for *cw* = 250 μs).

We have shown before (Figures 6A,B) that the main factor setting a lower limit on the *N* is the spike rate at the peak. A longer *cw* increased spike rate, and can therefore lower the minimal *N* to get acceptable simulations. For longer *cw* (50-150-250 μs), the mean ± *SD* of the minimal *N* per dataset decreased from 3.92 ± 1.62 to 2.84 ± 1.63, and further to 2.46 ± 1.45.

For the same reason, a longer *cw* could also extend the range of possible values for *thr_bin_*. Figures 12C,D shows the maximal values of *thr_bin_* for TB datasets, for a *cw* of 150 μs (panel C) and 250 μs (panel D). The range of maximal *thr_bin_* was extended to respectively 6 and 8, but it can be seen that in the majority of cases it was still limited to ~2–5, except when *thr_mon_* was raised to high levels.

Thus, a longer *cw* helped achieving higher rates and therefore allowed lower *N* and higher *thr_bin_*. This came at the cost of a decrease in *MD*. Another effect is that a longer *cw* inherently causes temporal integration and will ultimately limit binaural temporal sensitivity.

### Comparison between TB and AN fibers

Because TB fiber spikes display enhanced temporal structure relative to the AN (Joris et al., 1994a,b; Louage et al., 2005), the probability of spikes from separate inputs occurring in the same *cw* will be higher. Therefore we hypothesized that the *N* required to get a given number of output spikes is lower for TB fibers. We compared the lowest *N* that generated acceptable simulations for TB and AN datasets, using *cw* of 50 μs. As mentioned above (section Estimation of Minimal *N*), for *thr_bin_* = 2, the minimal *N* was slightly lower for TB fibers (mean ± *SD* 4.08 ± 1.68) than for AN fibers (4.17 ± 0.72), but the difference did not reach statistical significance (two sample *t*-test *p* = 0.876). For *thr_bin_* = 3 however, the difference increased, with mean ± *SD* for TB 7.5 ± 1.41 and for AN 9.1 ± 1.46 (two sample *t*-test *p* = 0.04). Therefore TB fibers can be seen as more robust than AN as input fibers to the binaural neuron: the necessary increase in *N* for a less than perfect coincidence detector (one that fails to detect a single spike on both sides coinciding, i.e., *thr_bin_* > 2) is larger for AN fibers than for TB fibers, because the latter have more tightly synchronized and more reproducible firing patterns. Therefore TB fiber inputs provide a larger safety factor for the detection of binaural coincidences: if a particular input spike is missed, there is a larger probability that there are others in the same *cw* present in order to reach spike threshold. This can be seen as well in the range of possible values for *thr_bin_*, for TB inputs vs. AN inputs, where less AN than TB fibers could fulfill a requirement of 3 binaural coincidences (compare Figures 12A,B).

**Figure 14:**
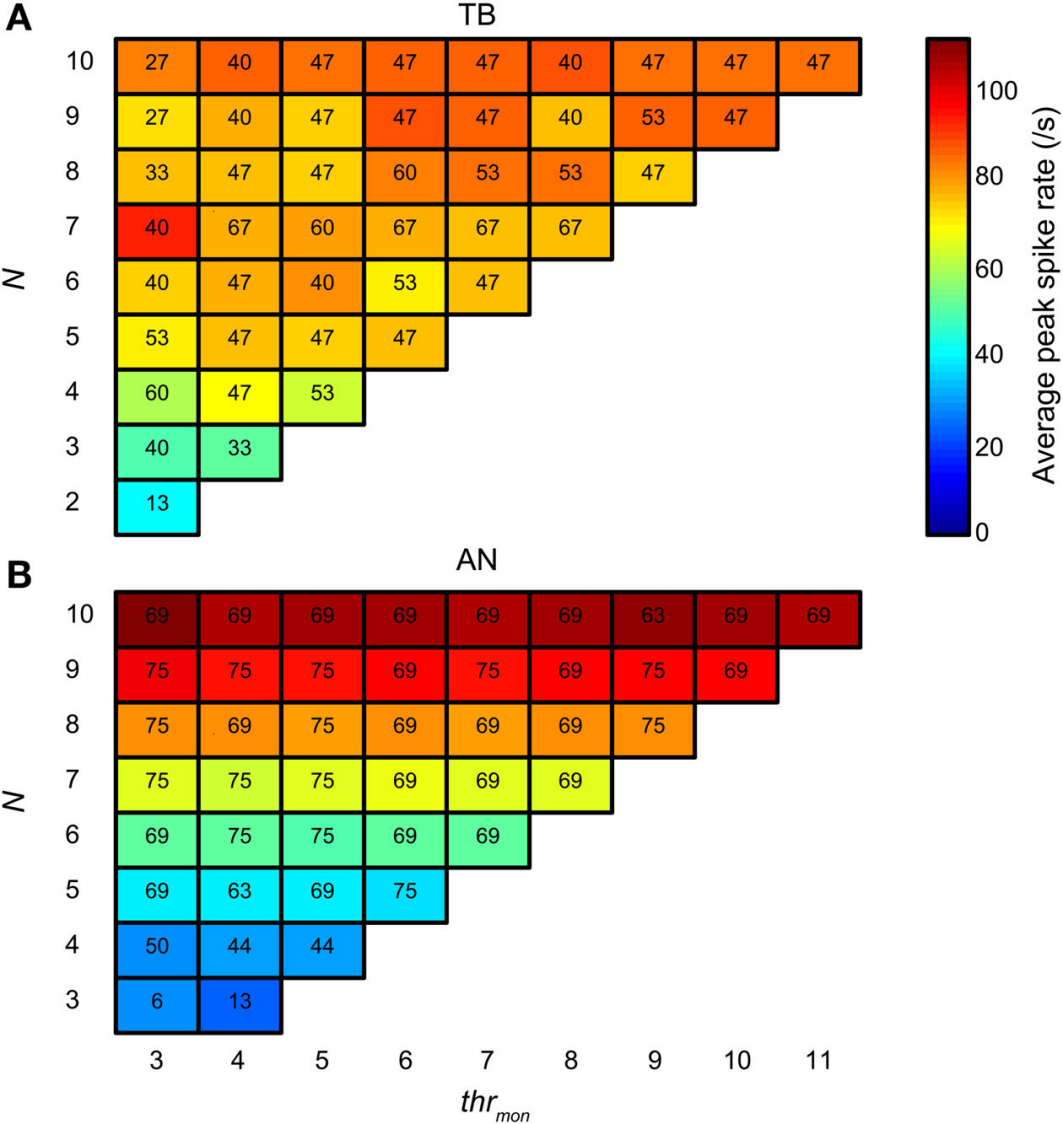
**Average spike rate at NDF peak for TB (A) and AN (B) simulations.** The color of each subplot indicates the average peak rate for accepted simulations with *thr_mon_* and *N* indicated respectively by the abscissa and ordinate. *cw* = 50 μs, and *thr_bin_* = 2. Color scale is the same in **(A)** and **(B)**. The number in each subplot indicates which proportion (in %) of the individual datasets had at least one accepted simulation for that combination of parameters.

Next we compared the simulation output for TB and AN datasets, for the same simulation parameters. Here, *thr_bin_* = 2. Figure 14 groups the accepted simulations for each combination of *N* (varied along the ordinate) and *thr_mon_* (varied along the abscissa), using a *cw* of 50 μs. The background color of each subplot indicates the average of the peak spike rate for all accepted simulations with those parameters. As expected, and in agreement with the examples in Figures 4, 5, it can be seen that the maximal spike rate increased with increasing *N*, in both TB (panel A) and AN (panel B). Comparing the two panels, we observe that for low *N*, i.e., 3–7, the peak rate was higher in the TB case than in the AN case (i.e., “cooler” colors lower half of figure for AN). Surprisingly, for *N* > 7, the peak rate of AN simulations was larger than of TB simulations: the gradient in spike rate with increasing *N* was much more distinct in the AN than in the TB. Thus, in addition to the other (coding) advantages that TB fibers have above AN fibers (see Discussion), TB fibers allow sparser connectivity than AN fibers: it takes less inputs to get similar spike rates, at least within the lower range of *N*, and the output spike rate is also less dependent on the exact *N*. This is again a consequence of enhanced synchronization, because it takes fewer inputs to get enough spikes to reach coincidence threshold when there is consistent spiking with low temporal jitter. Increasing the *N* even more hardly increases the spike rate further, because the number of spikes is limited to maximally one spike per *cw* and per effective stimulus cycle.

The number in each subplot of Figures 14A,B indicates the percentage of datasets contributing at least one accepted simulation for that set of parameters. It can be seen that for low *N* (2–4), relatively more TB fibers contributed good simulations than AN fibers. For higher *N*, the opposite was true.

**Figure 15:**
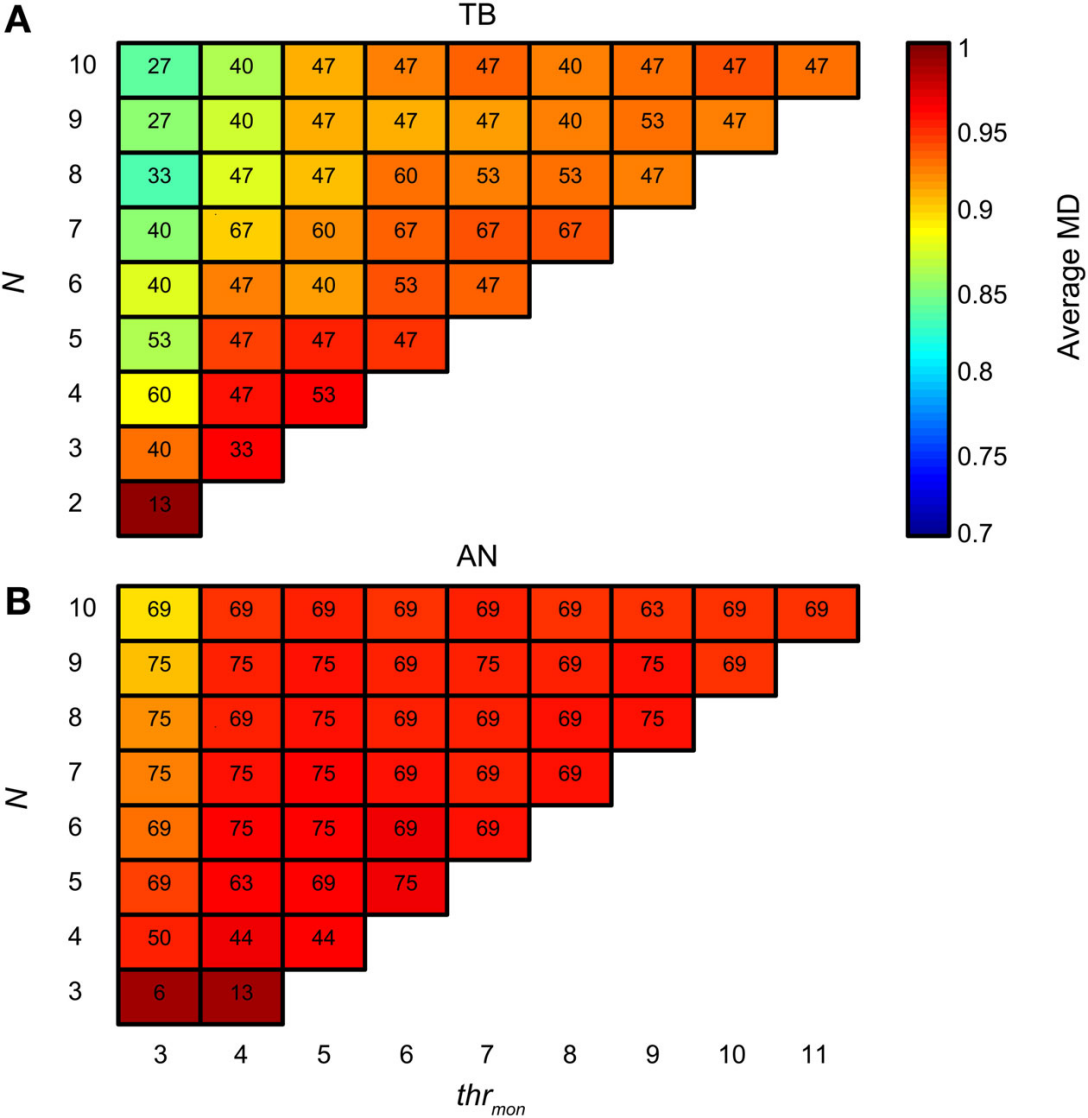
**Average NDF modulation depth for TB (A) and AN (B) simulations.** The color of each subplot indicates the average peak rate for accepted simulations with *thr_mon_* value and *N* indicated by the abscissa and ordinate. *cw* = 50 μs, and *thr_bin_* = 2. Color scale is the same in **(A)** and **(B)**. The number in each subplot indicates which proportion (in %) of the individual datasets had at least one accepted simulation for that combination of parameters.

There is also a downside of having tight and consistent synchronization: the proportion of monaural coincidences rises fast with *N*. Figure 15A is organized like Figure 14A, but now the colors indicate the average *MD* for accepted simulations with TB datasets. For low *thr_mon_* (e.g., 3 or 4), *MD* decreased rapidly with increasing *N*, due to a “DC-shift” (cf. Figure 4). This effect was counteracted by increasing *thr_mon_*. For simulations with AN datasets (Figure 15B), the average MD showed milder dependence on *N*. The different requirements for the minimal *thr_mon_* between AN and TB were already illustrated above (Figure 9).

Thus, TB fibers as inputs to the binaural coincidence detector are advantageous in that a low number of fibers can already result in a physiologically realistic output spike rate. The downside is that monaural coincidences will increase as well with more inputs, so that a mechanism to suppress the efficacy of monaural coincidences becomes more critical for an increasing number of well-synchronized inputs.

## Discussion

We studied the output of a binaural coincidence detector to experimentally derived input spike trains. Our approach differs fundamentally from existing MSO models. Instead of attempting to make a detailed biophysical model, we used a bare bones approach: the binaural neuron was reduced to the fundamental operation it performs on its excitatory inputs, i.e., an output spike was generated when input events occur close enough in time (Marsalek and Lansky, 2005; Jennings and Colburn, 2010; Sanda and Marsalek, 2012). The advantage is that a few conceptually simple and independently manipulated parameters (number of inputs *N*, sensitivity to monaural and binaural coincidences, and coincidence window *cw*) could be studied for their effect on the output, without violating the fundamental operation the neuron performs. This simplified parameter space limited the number of assumptions that have to be made. As an example, *cw* is determined by several parameters including synaptic event kinetics, voltage-activated potassium currents (Dasika et al., 2007; Mathews et al., 2010), inhibition (Roberts et al., 2013), post-inhibitory rebound (Brand et al., 2002; Sanda and Marsalek, 2012) and dendrites (Grau-Serrat et al., 2003). In our model, it was abstracted to a single parameter. However, our presynaptic stage was more realistic than the inputs used in previous modeling studies because we fed the coincidence detector with TB or AN spike trains recorded *in vivo*. Moreover this presynaptic stage was also more generic than in previous modeling studies because these spike trains were in response to broadband noise. The simulation output was compared to recordings from binaural neurons for its physiological plausibility. This allowed us to explore the effect of the parameter values on the output spike train. Despite its simplicity, this approach leads to several clear conclusions.

The results showed that convergence of typically ≥4 inputs on each side is needed for a realistic output. Especially for narrow *cw*, the neuron has to be very sensitive to single coinciding input events in order to generate enough output spikes. Monaural coincidences need to be suppressed more than binaural coincidences. Convergence of multiple inputs reduces the expansive relationship between correlation and output spike rate that is seen in TB recordings, but not to the extent that is found in actual binaural neurons. Increasing the *cw* allows higher values for *thr_bin_* and lower values for the *N*. TB fibers are advantageous to AN fibers in generating higher spike rates for low *N*.

The lower limit on *N* corresponds well with the estimation of minimally 2–4 excitatory inputs on each side in a recent *in vitro* study (Couchman et al., 2010). In reality the required number of excitatory inputs may be higher, given the fact that there are glycinergic inhibitory inputs converging on MSO neurons as well, most likely making it harder for the neuron to reach threshold than in the absence of inhibition (Cant, 1991; Cant and Hyson, 1992). This is supported by the fact that MSO spike rates increase with application of strychnine during *in vivo* recordings (Brand et al., 2002; Pecka et al., 2008). Simple estimates based on juxtacellular (van der Heijden et al., 2013) and intracellular (Franken et al., 2013) recordings of MSO in the gerbil, combined with data from estimates of spontaneous activity in presumed SBCs in the gerbil (Karino and Joris, 2009; Kuenzel et al., 2011) suggest a convergence of ~10 fibers (total, i.e., contra + ipsi). Anatomical data are required to narrow down this important parameter.

Given the suggested small *N*, our finding that the *thr_bin_* has to be very low is consistent with the simple view, first suggested by Jeffress (1948), of a coincidence process comparing the timing of individual spikes supplied by afferents from the two ears. This situation is conceptually different from that thought to occur in the barn owl, where binaural neurons receive a very large number of afferents [~45–150 from each side, (Carr and Boudreau, 1993)] and where the binaural comparison is more akin to the summation of two AC waveforms, described as the “sound analogue potential” (Funabiki et al., 2011; Ashida et al., 2012).

Perhaps the most surprising outcome of our study is that coincidence counts on physiologically recorded spike trains clearly point to the need for a higher monaural than binaural coincidence threshold, to suppress monaural coincidences. It has long been known that monaural response rates are typically lower than maximal binaural response rates (Goldberg and Brown, 1969; Yin and Chan, 1990). However, this observation in itself does not imply a difference in monaural vs. binaural coincidence threshold (Colburn et al., 1990). Our finding that *thr_mon_* has to exceed *thr_bin_*, strongly implies that an additional mechanism is necessary to promote binaural coincidences over monaural coincidences, in addition to the combinatorial effect suggested by Colburn et al. (1990). Suppression of the efficacy of monaural coincidences can be provided by the dendrites, through sublinear summation of events from the same side, and/or the opposite dendrite functioning as a dendritic sink (Agmon-Snir et al., 1998). A recent *in vivo* study with mainly juxtacellular recordings suggested that the current sink was not very large (van der Heijden et al., 2013). We demonstrate for the first time that the necessity of suppression of monaural coincidences (in addition to the combinatorial effect) directly follows from realistic cochlear nucleus inputs. This is surprising given the fact that multiple inputs per side in models with a single compartment (in which case *thr_mon_* does not differ from *thr_bin_*) have been able to reproduce realistic MSO rate ITD functions (Colburn et al., 1990; Han and Colburn, 1993; Brughera et al., 1996; Svirskis et al., 2003), be it to tones rather than to broadband noise. On the other hand, the argument of Colburn et al. might be more applicable to the barn owl nucleus laminaris because it receives vastly more inputs: this will result in a greater combinatorial effect, as explained in Results (compare *x* = 4 with *x* = 2 in Figure 10). An additional mechanism to suppress monaural coincidences might therefore not be needed in the owl. This reasoning seems to be supported by the short and stubby dendrites of laminaris neurons, especially in high frequency regions (Carr and Boudreau, 1993). An additional mechanism that may increase *MD*, and thereby counteract the effect of monaural coincidences, is the shunting effect of somatic low-voltage activated potassium currents (Grau-Serrat et al., 2003), which can further lower the spike rate for out-of-phase ITDs.

Another way of addressing the different treatment of monaural vs. binaural coincidences would have been to choose separate values for *cw* for monaural and binaural coincidences, instead of *thr_mon_* and *thr_bin_*. In a more general sense, a coincidence window can be regarded as having a certain width (corresponding to our parameter *cw*) and a certain height (corresponding to our parameters *thr_mon_* and *thr_bin_*). Because generating output spikes will be easier for either a shorter duration or a lower height of the coincidence window, *cw* and *thr* are to a certain extent interchangeable. Because separate *thr_mon_* and *thr_bin_* are biophysically easier to understand given sublinear dendritic summation (Agmon-Snir et al., 1998), we did not opt for different *cw_mon_* and *cw_bin_*.

Previous work from our laboratory showed that TB (pseudobinaural) correlation functions are highly expansive, whereas the sensitivity of IC neurons to interaural correlation is surprisingly shallow (Mc Laughlin et al., 2008, 2014). Convergence increases the probability of coincidences, and this effect will be greater for lower correlation values, because the refractory period puts an upper limit on the spike rate, and the number of output spikes in a coincidence window is maximally one. This increase in rate thus affects the “tail” of the rICF function, causing the power *p* to decrease. However we found that the power in this study is still significantly higher than for binaural neurons. This confirms the moderate decrease of power of the example TB dataset shown by Mc Laughlin et al. (2014) for 10 inputs per side when compared to single inputs. Additional factors that can decrease this expansiveness are jitter in the inputs (Mc Laughlin et al., 2014), a slightly different CF of inputs converging on the same neuron (Joris et al., 2006a) or differences in temporal microstructure between fibers with similar CFs. Unfortunately, we do not have sufficient TB data to systematically study the latter effects.

We varied *cw* from 50 to 250 μs. Larger coincidence windows allow lower *N* and higher *thr_bin_*, but increase the number of monaural coincidences and therefore will raise the minimal *thr_mon_*. As mentioned above (introductory paragraph of Discussion), *cw* is an abstract quantity that is determined by many cellular parameters. Various sources of information suggest that the chosen values are a realistic range for *cw*. The halfwidth of ITD tuning curves obtained *in vitro* is ~200–400 μs in mammal and chick (Joseph and Hyson, 1993; Kuba et al., 2003; Fischl et al., 2012; Roberts et al., 2013), which corresponds to a *cw* ~ 100–200 μs. It is unclear to what extent these estimates apply to the *in vivo* situation. Second, fine-structure ITD sensitivity in cat goes up to almost ~3 kHz (Rose et al., 1966; Yin and Kuwada, 1983; Joris and Verschooten, 2013), which has a period of 333 μs, thus *cw* can only be a fraction of that value. At present it is unclear whether *cw* differs for neurons with different CF. One study reports a gradient in intrinsic membrane properties along the tonotopic axis (Baumann et al., 2013), but this decreases during development and has not been found in another study (Scott et al., 2007). Third, previous work in our lab has shown that *cw* = 50 μs is short enough to describe the temporal microstructure in TB fibers (Joris et al., 2006b). Since MSO cells are the fastest cells targeted by TB fibers, it is reasonable to assume that they are able to detect this microstructure. Some computational models have used even lower values for the *cw* (e.g., 20 μs Krips and Furst, 2009). A previous black-box model has shown that spike rate increases when *cw* broadens (Sanda and Marsalek, 2012), but in their case only a single input—modeled on the AN—contacted the coincidence detector. This was not the case in a biophysical model of the barn owl NL, where slower synaptic events decrease spike rate due to a decrease in the sound-analogue potential (Ashida et al., 2013).

TB fibers are advantageous relative to AN fibers in this coincidence scheme with converging inputs, because they are more resistant to higher *thr_bin_*, and achieve higher spike rates for low *N* with spike rate being less dependent on *N*: all of which are consequences of increased synchronization with decreased temporal jitter and higher trial-to-trial reliability. This advantage may be even larger in the presence of inhibition, which is not accounted for in our coincidence scheme. A previous modeling study found that modeled high-sync (TB) inputs resulted in ITD functions with more realistic spike rates and synchronization index compare to functions obtained with modeled AN inputs, even though a point neuron model was used, thus disregarding differential effects of monaural and binaural coincidences (Brughera et al., 1996).

Although our presynaptic stage is inherently more physiological than any existing input model, it is still far from being realistic. A first limitation is that we did not incorporate the inhibitory inputs to MSO, which have been proposed to play a critical role in ITD processing (Brand et al., 2002; Pecka et al., 2008). The presence of inhibition may necessitate an increase in the minimal number of excitatory inputs required, but too little is known about the inhibition to make strong predictions. Similarly, *thr_bin_* may need to be lower in the presence of inhibition. A second limitation is that we used TB responses without knowing whether they were recorded from SBCs or GBCs. Thus, it is possible that in some cases we were applying inputs that are in fact inhibitory onto MSO as excitatory inputs. Physiological differences between low-CF SBCs and GBCs are at present unclear, as has been commented on in previous publications from our laboratory (Joris and Smith, 2008). Additional single fiber labeling experiments are needed to clarify this point. A third limitation is that in the simulations presented here, all inputs are derived from the same TB axon. Unfortunately, present recording techniques do not allow the dense sampling needed to record a small population of TB axons centered near the same CF in a single animal. Even for AN recordings, such sampling is difficult to achieve (Sachs and Young, 1979; Delgutte, 1980; Joris et al., 2006a). Nevertheless it is safe to say that the actual inputs converging on an MSO neuron are bound to differ more from each other than in our simulations, where the inputs are all derived from the same neuron. Future recordings can alleviate the problem of lacking recordings with similar or close CFs partially by obtaining recordings from one neuron to time-warped stimuli (Heinz, 2007; Heinz et al., 2010). However, the larger question—how similar or dissimilar are inputs to an MSO neuron?—will require substantially more refined techniques than are presently available. Two of our main observations (the requirement for a higher *thr_mon_* than *thr_bin_*, and for a low *thr_bin_* to obtain enough output spikes) apply to both AN responses and TB responses as inputs. Because spike trains of AN fibers are less stereotyped than in TB fibers, this suggests that our observations will also hold for the *in vivo* situation, where non-identical SBCs converge on an MSO neuron.

Of course much more limitations can be pointed out for the output stage. A limitation at present is that our knowledge of MSO responses is still limited, and this is certainly the case for responses to broadband noise. Besides a few examples recorded by Yin and Chan (1990) and the recordings, used here, from axons of presumed MSO neurons (Bremen and Joris, 2013), we relied heavily on the more extensive data in IC neurons in response to noise, particularly regarding the responses to changes in ρ (Yin et al., 1987; Shackleton et al., 2005; Coffey et al., 2006; Joris et al., 2006a,b; Mc Laughlin et al., 2008).

We conclude that a simple coincidence analysis of monaural broadband noise recordings predicts that a small number of inputs converge on MSO neurons, that these neurons act as coincidence counters on a very low number of spikes, as envisaged by Jeffress (1948), and that this scheme requires a mechanism to suppress monaural coincidences in addition to the combinatorial explanation of Colburn et al. (1990).

## Author contributions

Tom P. Franken, Peter Bremen, and Philip X. Joris designed the study. Tom P. Franken performed research. Tom P. Franken, Peter Bremen, and Philip X. Joris wrote the paper.

### Conflict of interest statement

The authors declare that the research was conducted in the absence of any commercial or financial relationships that could be construed as a potential conflict of interest.

## Abbreviations

AC: autocorrelogram
AN: auditory nerve
BW: bandwidth
CF: characteristic frequency
*cw*: coincidence window
DF: dominant frequency
GBC: globular bushy cell
ITD: interaural time difference
LL: lateral lemniscus
*MD*: modulation depth
MSO: medial superior olive
*N*: number of inputs per side
NDF: noise-delay function
rICF: rate interaural correlation function
SBC: spherical bushy cell
SD: standard deviation
SR: spontaneous rate
*thr_bin_*: binaural coincidence threshold
*thr_mon_*: monaural coincidence threshold
TB: trapezoid body.
